# Leveraging a neutrophil-derived PCD signature to predict and stratify patients with acute myocardial infarction: from AI prediction to biological interpretation

**DOI:** 10.1186/s12967-024-05415-0

**Published:** 2024-07-02

**Authors:** Yihao Zhu, Yuxi Chen, Yao Zu

**Affiliations:** 1 International Research Center for Marine Biosciences, Ministry of Science and Technology, Shanghai Ocean University, Shanghai, 201306 People’s Republic of China; 2 Key Laboratory of Exploration and Utilization of Aquatic Genetic Resources, Ministry of Education, Shanghai Ocean University, Shanghai, 201306 People’s Republic of China; 3 Marine Biomedical Science and Technology Innovation Platform of Lin-Gang Special Area, Shanghai, 201306 People’s Republic of China

**Keywords:** Programmed cell death, Neutrophil, Machine learning, Diagnosis, Mechanism

## Abstract

**Background:**

Programmed cell death (PCD) has recently been implicated in modulating the removal of neutrophils recruited in acute myocardial infarction (AMI). Nonetheless, the clinical significance and biological mechanism of neutrophil-related PCD remain unexplored.

**Methods:**

We employed an integrative machine learning-based computational framework to generate a predictive neutrophil-derived PCD signature (NPCDS) within five independent microarray cohorts from the peripheral blood of AMI patients. Non-negative matrix factorization was leveraged to develop an NPCDS-based AMI subtype. To elucidate the biological mechanism underlying NPCDS, we implemented single-cell transcriptomics on Cd45+ cells isolated from the murine heart of experimental AMI. We finally conducted a Mendelian randomization (MR) study and molecular docking to investigate the therapeutic value of NPCDS on AMI.

**Results:**

We reported the robust and superior performance of NPCDS in AMI prediction, which contributed to an optimal combination of random forest and stepwise regression fitted on nine neutrophil-related PCD genes (MDM2, PTK2B, MYH9, IVNS1ABP, MAPK14, GNS, MYD88, TLR2, CFLAR). Two divergent NPCDS-based subtypes of AMI were revealed, in which subtype 1 was characterized as inflammation-activated with more vibrant neutrophil activities, whereas subtype 2 demonstrated the opposite. Mechanically, we unveiled the expression dynamics of NPCDS to regulate neutrophil transformation from a pro-inflammatory phase to an anti-inflammatory phase in AMI. We uncovered a significant causal association between genetic predisposition towards MDM2 expression and the risk of AMI. We also found that lidoflazine, isotetrandrine, and cepharanthine could stably target MDM2.

**Conclusion:**

Altogether, NPCDS offers significant implications for prediction, stratification, and therapeutic management for AMI.

**Supplementary Information:**

The online version contains supplementary material available at 10.1186/s12967-024-05415-0.

## Introduction

Acute myocardial infarction (AMI) is the most severe manifestation of coronary artery disease (CAD), which is defined pathologically as myocardial necrosis resulting from ischemia and hypoxia caused by occlusion of coronary arteries [1]. Owing to its high morbidity and mortality, timely detection of AMI is imperative to guide more appropriate prevention and individualized treatment [2]. Given the low sensitivity and specificity of traditional electrocardiographic (ECG) in diagnosing AMI, the upturn of more sensitive cardiac biomarkers, preferably cardiac troponin (cTn) as the gold-standard biomarker, has been introduced and is widely engrained in the clinical practice of AMI identification [2, 3].

The joint European Society of Cardiology (ESC) and the American College of Cardiology (ACC) have proposed a biomarker-guided approach to redefine AMI diagnosis [4]. A patient labeled as AMI denotes the presence of myocardial injury detected by a characteristic abnormal pattern of cardiac biomarkers in the setting of evidence of acute myocardial ischemia. Nonetheless, these currently available biomarkers (represented by cTn) have gained glaring diagnostic performance in terms of high assay sensitivity, but at the cost of declining specificity [3, 5]. Worthy of note is that elevation in cardiac biomarkers could not differentiate tissue injury in AMI from other pathophysiological conditions, such as non-acute coronary syndrome (ACS) conditions and chronic diseases [3, 6], suggesting the achievement of heart-specificity rather than disease-specificity. The non-specific elevation in the presence of disruptive pathophysiological conditions has become a troubling problem [6].

Current AMI biomarkers are far from ideal, so our exploration of novel biomarkers with high sensitivity and specificity will never end [7]. Analysis of these currently conventional biomarkers has not contributed considerably to adding more diagnostic value since limited predictive information representing a single pathobiological process was obtained [8]. As such, a multi-biomarker assay strategy has been recommended to improve better diagnosis, risk stratification, and tailored treatment [9]. Over the past decades, significant advancements in bioinformatics techniques have been achieved, facilitating the generation of massive biomedical data for exploration [9, 10]. With the burgeoning interest in rapidly mining novel molecular biomarkers leveraging bioinformatics-based approaches, several reports on surveying AMI biomarkers have emerged [11–15]. Disappointingly, many of these studies were irrationally implemented by merging several AMI datasets from distinct origins (peripheral blood, peripheral blood mononuclear cells, thrombosis, endothelial cells, etc.), which hampered the reliability of conclusions because of the lack of tissue-specificity. Of greater concern, reasonable interpretation of the molecular mechanisms of biomarkers has generally been lacking or limited in work to date, which is detrimental to our understanding of the interrogate pathogenesis and progression of AMI. Concerning these shortcomings, we curated peripheral blood, which offers rapid and effective detection [16], as the single-source specimen to guarantee reliability and generalizability. We also proposed a multimethod-driven interpretation to unveil the mechanisms underlying biomarkers in AMI progression.

Neutrophils, the most abundant leukocyte type in peripheral blood, are recruited early in ischemic myocardial tissues after infarction within the first few hours post-AMI [17]. Recruited neutrophils initially intend to coordinate death cell debris removal, contributing to cardiac repair [18]. Nevertheless, excessive infiltration or delayed regression of neutrophils releases an abundant production of cytokines, chemokines, and reactive oxygen species (ROS), translating into aggravation of cardiac damage [17, 19]. Subsequently, neutrophil reactions post-AMI should be tightly regulated. Evidence shows that programmed cell death (PCD), particularly apoptosis and NETosis, is pivotal to the balance between neutrophil infiltration and removal post-AMI [20]. Still, more information is needed about the roles or dynamics of neutrophil-derived PCD in AMI, which sparked our great interest in revisiting this signature on the contribution of diagnosis and mechanisms. To fill these knowledge gaps, we herein hypothesized the utility of neutrophil-derived PCD signature as “sentinel” detection of AMI.

**Fig. 1:**
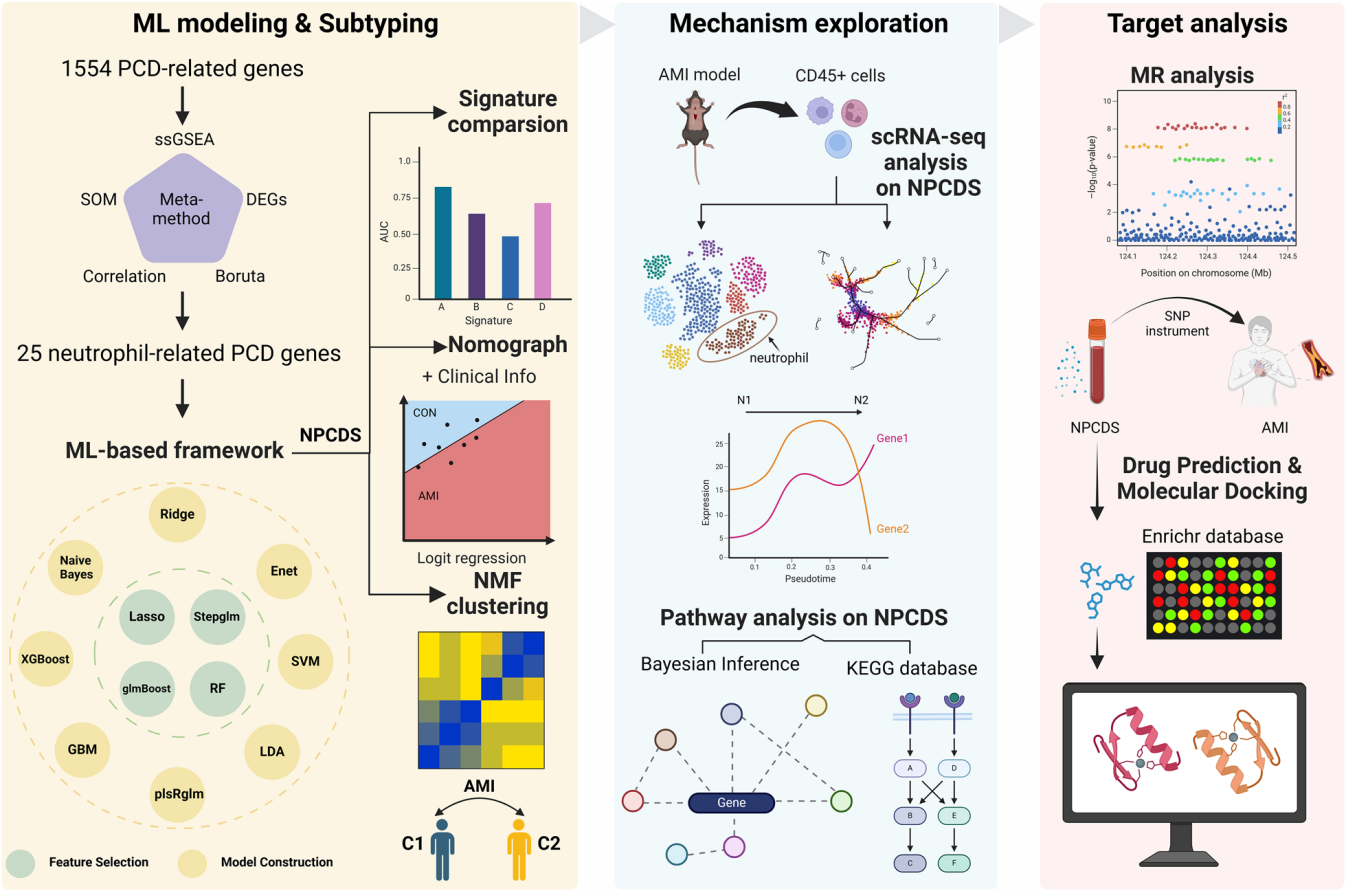
The overall analysis flow of this study. The flow can be divided into three aspects: ML modeling and subtyping, mechanism exploration, and target analysis (Fig. 1 was created with BioRender.com with premission)

The overall design of this study is depicted in Fig. 1. Gene expression profiles from microarrays were collected from the peripheral blood of AMI patients. We initially gathered a panel of reliable neutrophil-related PCD genes, rather than a single biomarker, for the subjection to an integrative machine learning (ML)-based framework. Upon benchmarking and evaluation, we then introduced a novel and robust model for AMI prediction, termed the neutrophil-derived PCD signature (NPCDS). Leveraging non-negative matrix factorization (NMF) on NPCDS, two distinct molecular subtypes of AMI were subsequently identified. Performing scRNA-seq analysis on Cd45+ immune cells isolated from murine hearts with AMI and Bayesian network inference on NPCDS-modulated pathways, we rationally interpreted the biological mechanisms underlying NPCDS across neutrophil transformation in AMI. We also conducted a two-sample Mendelian randomization (MR) study, combined with molecular docking, to investigate the causal relationship of NPCDS with AMI and its drugability. Overall, our novel findings offer a well-performance NPCDS for detecting and stratifying patients with AMI, which may benefit precision diagnosis and individualized treatment.

## Methods and materials

### Data collection

Gene expression profiles of peripheral blood derived from AMI patients, including eight independent cohorts (GSE123342, GSE29532, GSE60993, GSE61144, GSE97320, GSE48060, GSE194388, and GSE34198), were retrieved from the GEO database (www.ncbi.nlm.nih.gov/geo). Among these cohorts, five independent cohorts (GSE123342, GSE29532, GSE60993, GSE61144, and GSE97320) [21–24] were utilized to develop a Neutrophil-related PCD Signature (NPCDS). GSE48060, GSE194388, and GSE34198 were selected as external validation cohorts to assess the predictive performance of NPCDS. GSE34198 [25], which contained clinical information on AMI patients, was also selected to develop a nomogram incorporating clinical information and NPCDS. The raw data from the array-based method was all processed after quantile normalization and log2 transformation, which could be used for downstream analysis. To explore the mechanism of NPCDS in AMI progression, we gathered GSE163465 [26], a single-cell RNA sequencing (scRNA-seq) dataset to map Cd45+ immune cells isolated from mice left LAD ligation. To deepen our understanding of NPCDS on neutrophils, GSE151571 was enrolled, as it contained heart specimens from Mdm2 knockout (heart-specific) mice [27]. The detailed information on these enrolled data is summarized in Table 1.

**Table 1:** Information of AMI-related datasets enrolled in study

GSE accession	Sample source	Platform	Sequencing type	Control samples	AMI samples	Cohort usage
GSE123342	Peripheral blood	GPL17586	Array	22	65	Training cohort
GSE29532	Peripheral blood	GPL5175	Array	6	8	Testing cohort
GSE60993	Peripheral blood	GPL6884	Array	7	17	Testing cohort
GSE61144	Peripheral blood	GPL6106	Array	10	7	Testing cohort
GSE97320	Peripheral blood	GPL570	Array	3	3	Testing cohort
GSE48060	Peripheral blood	GPL570	Array	21	31	External validation cohort
GSE194388	Peripheral blood	GPL24676	RNA-seq	5	10	External validation cohort
GSE34198	Peripheral blood	GPL6102	Array	48	49	External validation and nomogram development cohort
GSE163465	Cd45^+^ cell	GPL24247	scRNA-seq	1	3	scRNA-seq cohort
GSE151571	heart	GPL21103	RNA-seq	3 (Control group with Mdm2 retainment)	5 (Mdm2 knockout group)	In-vivo experimental cohort

### Identification of neutrophil-related PCD genes

The GSE123342 dataset, which contained 65 AMI and 22 control cases, was selected as the training cohort for identifying neutrophil-related PCD genes for NPCDS construction. We gathered a list of genes related to 17 PCD patterns (n = 1554; duplicate genes were removed) from a comprehensive literature retrieval [28], including apoptosis (n = 580), pyroptosis (n = 52), ferroptosis (n = 88), autophagy (n = 367), necroptosis (n = 101), cuproptosis (n = 19), parthanatos (n = 9), Entotic cell death (n = 38), NETosis (n = 32), lysosome-dependent cell death (LDCD) (n = 220), alkaliptosis (n = 7), oxeiptosis (n = 5), immunogenic cell death (n = 34), anoikis (n = 338), paraptosis (n = 66), methuosis (n = 8), and disulfidptosis (n = 9). We also collected the neutrophil-related gene sets (n = 39) [29]. The detailed gene list can be found in Supplementary Tables 1 and 2. Based on the PCD-related gene sets, single sample gene set enrichment analysis (ssGSEA) implemented in the R package “GSVA” (version 1.48.0) [30] was performed on the expression profile to assess the active PCD patterns enriched in AMI. Using the GSE123342 dataset, neutrophil-related PCD genes were identified according to the following meta-method process:

- We employed the R package “kohonen” (version 3.0.11) [31] to perform a Self-organizing map (SOM) on the expression profile of AMI cases. Expression values of the gene were converted into Z-score. A featured map was generated with a definition as a 10 × 10 grid for input. Gene patterns with similar expressions were assigned to adjacent clusters after hierarchical clustering on SOM-identified nodes (Supplementary Fig. 1), and clusters with highly expressed gene sets were retained.
- Differentially expressed genes (DEGs) between AMI and control cases were analyzed through the R package “limma” (version 3.56.1) [32]. The cut-off criteria for DEGs were set as: |log_2_Fold Change (FC)|> 0.6 and false discovery rate (FDR) < 0.05. We preserved the up-regulated DEGs labeled with log_2_FC greater than 0.6 and FDR less than 0.05.
- Pearson’s correlation coefficients between active PCD pattern-related genes and neutrophil-related genes were calculated via the R basic function “cor.test”. We retained PCD-related genes with a R value greater than 0.5 and a P value less than 0.001.
- Taking the intersection genes of the three analyses above, we obtained a list of neutrophil-related PCD genes, demonstrating a strong correlation with neutrophil-related gene expressions and an up-regulation pattern in AMI.
- We adopted the R package “Boruta” (version 8.0.0) [33] to execute feature selection on the neutrophil-related PCD genes. To ensure the robustness of feature selection, we set the parameters of running Boruta as maxRuns = 10,000, pValue = 0.01, and mcAdj = TRUE, which indicated a bootstrap-based selection [34]. After comparing the importance of neutrophil-related PCD genes and maximum shadow ATTRIBUTE, we retained Boruta-accepted features, which were considered highly relevant to AMI.
- Finally, we gathered a list of 25 neutrophil-related PCD genes after implementing the Boruta algorithm. To further unravel the biological processes and signaling pathways of these genes, the R package “DOSE” (version 3.26.1) [35] and “clusterProfiler” (version 4.8.1) [36] were utilized to carry out Disease Ontology (DO), Gene Ontology (GO), and Kyoto Encyclopedia of Genes and Genomes (KEGG) enrichment analyses. Enriched terms with a Q-value less than 0.05 were regarded as significant.

### Construction of neutrophil-related PCD signature (NPCDS) based on an integrated ML-based framework

We curated 25 neutrophil-related PCD genes in the previous meta-method analysis. Through overlapping genes in the training and four independent testing cohorts (GSE29532, GSE60993, GSE61144, and GSE97320), namely genes with probes in each cohort, 20 neutrophil-related PCD genes were retained. To establish a stable and accurate Neutrophil-related PCD Signature (NPCDS), we benchmarked 12 ML algorithms. Then, we applied them to construct an integrative framework based on the expression profiles of 20 neutrophil-related PCD genes from independent cohorts (GSE123342, GSE29532, GSE60993, GSE61144, and GSE97320). ML algorithms enrolled in this integrative framework included least absolute shrinkage and selection operator (Lasso), Ridge, elastic network (Enet), stepwise generalized linear model (Stepglm), support vector machine (SVM), generalized linear model by likelihood-based boosting (glmBoost), linear discriminant analysis (LDA), partial least squares regression for generalized linear models (plsRglm), Random Forest (RF), gradient boosting machine (GBM), eXtreme Gradient Boosting (XGBoost), and NaiveBayes [37, 38]. The R packages “randomForestSRC” (version 3.2.2) [39], “glmnet” (version 4.1-7) [40], “plsRglm” (version 1.5.1) [41], “gbm” (version 2.1.8.1) [42], “caret” (version 6.0-94) [43], “e1071” (version 1.7-13) [44], “BART” (version 2.9.4) [45], “MASS” (version 7.3-58.4) [46], “xgboost” (version 1.7.5.1) [47] were used to execute these ML algorithms. The procedure for generating NPCDS was described in the following pipeline:

- Before establishing NPCDS, we first performed a Z-score transformation [37] on the expression profiles of five independent cohorts, which could enhance comparability between diverse cohorts and accelerate running speed.
- The NPCDS was first discovered and generated in the training cohort. In this integrative ML-based framework, four algorithms with feature selection capability (Lasso, RF, Stepglm, glmBoost) were initially conducted to narrow down 20 neutrophil-related PCD genes. Then, we adopted eight other algorithms (Ridge, Enet, SVM, LDA, plsRglm, GBM, XGBoost, NaiveBayes) to fit prediction models based on the genes selected via the four algorithms, respectively. In total, 113 model combinations were used to tune hyperparameters and fit models under tenfold cross-validation (CV).
- All model combinations were tested in four independent cohorts (GSE29532, GSE60993, GSE61144, GSE97320). For the performance evaluation of each model, the Area Under Curve (AUC) score across these cohorts was computed. The model with the highest average AUC within training and testing cohorts was considered optimal. And this optimal model was called Neutrophil-related PCD Signature (NPCDS), which was a combination of RF and Stepglm algorithms using nine genes (PTK2B, MAPK14, IVNS1ABP, MDM2, GNS, MYH9, TLR2, MYD88, CFLAR).

### Comparison of NPCDS with the published signatures of AMI prediction

To compare the prediction power of our NPCDS with other signatures, we retrospectively conducted a literature retrieval. Herein, we focused on collecting mRNA expression signatures that predict AMI. Ultimately, a total of 63 signatures were enrolled in this comparative analysis, which can be found in Supplementary Table 7. These signatures were generated through various ML algorithms in the previous research, such as elastic net, Lasso, artificial neural network, and glmBoost. Additionally, these signatures reflect diverse AMI-related pathologic mechanisms, including the plaque rupture process and immune response. We excluded signatures where more than 30% of features were not matched in our collected cohorts. The AUC score was then calculated to compare the predictive performance between NPCDS and other public signatures.

### Development of a nomogram using NPCDS and clinical information

The GSE34198 dataset was selected as an external validation cohort for NPCDS. Additionally, it contains clinical information collected from patients with AMI, which could be integrated into a nomogram coupled with NPCDS scores. Clinical information includes continuous variables (Age, Height, Weight, SBP, DBP) and categorical variables (Diabetes status, Smoking status, ACEI, Betablockers, Diuretics, Ca2+ blockers, Statins, and Fibrates). A detailed record of the clinical information can be found in Supplementary Table 8. To construct and validate a nomogram, GSE34198 was first randomly classified into a GSE34198-training cohort and a GSE34198-testing cohort in a ratio of 6:4. We then employed NPCDS to yield a predicted score for each case. Next, we utilized the R package “rms” (version 6.7-1) [48] to carry out multivariate logistic regression using the NPCDS score and clinical information in the training cohort. Clinical characteristics with a P-value less than 0.05 in the regression analysis were retained to develop a nomogram. Leveraging multivariate logistic regression on fitting NPCDS scores, betablockers, ACEI, diabetes status, and statins, we finally established a graphic predictive nomogram. To systemically assess the precision of nomogram prediction, we performed the AUC score, calibration curve, and Decision Curve Analysis (DCA) analyses within the GSE34198-training cohort and GSE34198-testing cohort.

### Establishment of molecular subtypes of AMI using NPCDS

The R package “NMF” (version 0.26) [49] was employed to divide AMI patients into different subtypes using expressions of NPCDS. More than 15 AMI cases in the GSE123342, GSE60993, and GSE34198 datasets were included in NMF clustering to ensure stable identification. The “brunet” approach was selected with a setting of 100 iterations to execute NMF clustering. To determine the optimal number of clustering, we computed four indicators (cophenetic, residuals, RSS, silhouette) with a rank range of 2 to 10. The optimal number of clustering was set as 2 according to the cophenetic metrics, which robustly constructed two subtypes (Supplementary Table 9). The PCA plot and heatmap were then generated to assess the distribution and NPCDS expression between the two identified subtypes. In addition, we investigated the enriched differences of PCD, neutrophils, neutrophil degranulation, and neutrophil extravasation-related gene sets between the two proposed subtypes using the R packages “GSVA” (version 1.48.0) [30] and “clusterProfiler” (version 4.8.1) [36].

### Pre-processing of scRNA-seq analysis

GSE163465 was recruited as a scRNA-seq analysis dataset in this study, which provided the expression profiles of Cd45+ cells isolated from mice left sham surgery (control) and LAD ligation at different time points (AMI at 3-day, 7-day, and 14-day). First, quality control was performed on the raw count data using the R package “Seurat” (version 4.3.0) [50]. We filtered out cells with less than 200 or larger than 5000 feature counts and mitochondrial proportions larger than 20% [26]. The function “LogNormalize” was then utilized to normalize the filtered data. Second, the top 2000 highly variable genes between cells were identified via the function “FindVariableGenes”. The normalized data was subjected to a scaling process using the function “ScaleData” for the removal of variances. Third, principal component analysis (PCA) was applied to the normalized data. We selected the first 15 significant principal components (PCs) as the optimal dimension for subsequent t-distributed Stochastic Neighbor Embedding (t-SNE).

### Cell type annotation and Pseudotime analysis

Cells were clustered via the function “FindClusters”. Subsequently, the function “RunTSNE” was utilized to operate t-SNE based on the selected 15 dimensions. To highlight the role of NPCDS in immune cells, we removed clusters with lower Cd45 expression in the latter analysis. The identities of each cluster were annotated based on known gene markers [26]. Briefly, we annotated five cell populations: (1) Macrophage/Monocyte, as marked with higher expressions of C1qa, C1qb, C1qc, Adgre1, Ly6c2, Ccr2, Csf1r, and Cd68; (2) T/NK cell, as identified with higher expressions of Trbc2, Cd3d, Cd3e, Cd3g, Cd4, Cd8b1, Klra8, Klrb1c, Klrc1, Ncr1, Klra4, Klrc2; (3) Neutrophil, which demonstrated higher expressions of S100a9, S100a8, Lcn2, and Cxcl2; (4) B cell, as annotated via higher expressions of Cd79a, Cd79b, Ly6d, and Ebf1; (5) Dendritic cell, which showed higher expressions of Tmem123, Ccr7, Ccl22, Fscn1, Siglech, and Tcf4.

Next, we analyzed the numbers of each cell population among control, AMI at 3-day, AMI at 7-day, and AMI at 14-day to reveal the changes of immune cells in AMI dynamics (Supplementary Tables 11 and 12). Then, we analyzed the neutrophil population by repeating the dimensionality reduction and clustering analysis described above. Neutrophils were further annotated into two subtypes using the reported gene markers [26]: (1) N1, as reflected with higher expressions of proinflammatory genes such as Ccl2, Ccl7, Ccl9, Isg15, Ifitm6, Mmp8; (2) N2, as reflected with higher expressions of apoptosis or anti-inflammatory genes such as Sod2, Mif, Bnip3, Lgals3, Vim, and Il10. We then investigated the expression distribution of NPCDS among the N1 and N2 subtypes. To understand the molecular characteristics between diverse neutrophils, we applied the R package “AUCell” (version 1.22.0) [51] to calculate AUCell scores of NPCDS, neutrophils, and neutrophil degranulation to each neutrophil within the N1 and N2 subtypes. Additionally, we performed pseudotime analysis to uncover the differentiation trajectory of neutrophils using the R package “monocle3” (version 1.3.4) [52], with the previously normalized data as the input. The inferred trajectory was then projected into a two-dimensional UMAP plot. Based on Moran’s I test, we also gathered DEGs that varied in expression across the trajectory (Moran’s I > 0.01 and Q-value < 0.05). Using the R package “ClusterGVis” (version 0.1.1) [53], distinct kinetic patterns of DEGs across the trajectory were further determined by K-means clustering (Supplementary Table 13, Supplementary Fig. 7). The GO and KEGG terms of different gene patterns were also investigated using the R package “clusterProfiler” (version 4.8.1). Terms with a P-value less than 0.05 were significant enrichments of DEGs.

### Pathway analysis of NPCDS based on the KEGG database and Bayesian network

To gain insight into the molecular mechanism of NPCDS in AMI progression, we retrieved the NPCDS-regulated pathways from the KEGG database. Then, we performed a Bayesian network (BN) to deduce a gene regulatory network (GRN). The known pathways participated by NPCDS include neutrophil extracellular trap formation, Toll-like receptor signaling pathway, p53 signaling pathway, apoptosis, lysosome, and leukocyte transendothelial migration, as summarized in Supplementary Fig. 8. Next, we leveraged the R package “CBNplot” (version 0.99.2) [54], a BN-based computation framework, to infer a global GRN using the expressions of NPCDS and its interactive genes in known pathways.

### Two-sample MR analysis for the association between MDM2 and AMI

We sought to explore the causal effect of MDM2 (exposure) on AMI (outcome) using two-sample Mendel Randomization (MR). The R package “TwoSampleMR” (version 0.5.7) [55] was used to carry out MR analysis. The exposure data (ID: prot-a-1873) and outcome data (ID: ebi-a-GCST90038610) were retrieved from the IEU Open GWAS data source (https://gwas.mrcieu.ac.uk/). Single Nucleotide Polymorphisms (SNPs) used as instrumental variables (IVs) in MR analysis should be subject to three assumptions [56]: (1) SNPs are closely related to exposure; (2) SNPs are independent of cofounders of exposure and outcome; and (3) SNPs only affect the outcome through exposure. Accordingly, the function “extract_instruments” was utilized to retain the SNPs that were significantly associated with MDM2 (P-value < 5 × 10^–6^) but not with AMI (P-value > 0.05) [57]. SNPs with linkage disequilibrium (LD) R2 larger than 0.01 within a cropping range of 10,000 Kb were excluded [57]. Next, the function “harmonise_data” was leveraged to harmonize the exposure and outcome data. Two-sample MR analysis was subsequently performed via the function “mr”. Five default methods were adopted, including MR Egger, Weighted median, Inverse variance weighted (IVW), Simple mode and Weighted mode. The R package “forestploter” (version 1.1.1) [58] was employed to generate a forest plot showing the Wald ratio method-estimated odds ratio (OR) of MDM2 on AMI. The IVW approach, with the highest statistical power [56, 57], was selected to illustrate the association between the level of MDM2 and the risk of AMI. We employed heterogeneity, pleiotropy, and leave-one-out tests as sensitivity analyses, as well as reliability evaluations of MR. The heterogeneity test was performed to verify no heterogeneity in the analysis. Pleiotropy and leave-one-out sensitivity tests were conducted to assess whether SNPs affect MDM2 independently of AMI.

### Candidate drug prediction and molecular docking

Measuring the drug-protein interactions helps us understand how a gene could be developed as a drug target [59]. The small-molecule drugs interacting with MDM2 were predicted via the Enrichr database (https://maayanlab.cloud/Enrichr/) [60], which can be found in Supplementary Table 15. The top 5 drugs with high combined scores were selected, including ruthenium, lidoflazine, paclitaxel, isotetrandrine and cepharanthine. Regarding the cardiotoxicity of ruthenium and paclitaxel on patients with AMI [61, 62], we only retained lidoflazine, isotetrandrine, and cepharanthine as candidate small-molecule drugs targeting MDM2. Molecular docking, a computational approach for exploring the interaction between receptors and ligands [59], was implemented to evaluate the binding affinities of candidate drugs targeting MDM2. AutoDock Vina (version 1.1.2) software was utilized to execute molecular docking [63]. The analysis flow was as follows:

- The 2D structures of molecule ligands were downloaded from the PubChem database (https://pubchem.ncbi.nlm.nih.gov/) in SDF format. The ChemBio3D was performed to minimize energy on molecule ligands and export their 3D structures (mol2 format). AutoDock Vina then converted the molecule ligands (mol2 format) into PDBQT format for further docking analysis.
- The 3D structure of the protein receptor (MDM2) was passed through the Protein Data Bank (PDB) (https://www.rcsb.org/) from accession number 1RV1 [64]. We used PyMOL (version 2.4.0) to remove water molecules and ligands from proteins. Furthermore, the protein receptor (PDB format) was imported into the AutoDock Vina, which was exported as PDBQT format after polar hydrogenation and docking site setting.
- The parameters of the docking site, including X–Y–Z coordinates and grid size, were adjusted to include the active pocket that completely binds molecule ligands.
- AutoDock Vina was executed to dock the molecule ligands and protein receptor 20 times. Confident docking results with the lowest binding affinity and Root Mean Squared Error (RMSE) lower than 2 Å were retained and subsequently visualized by PyMOL.

### Analysis of neutrophil-related activities in Mdm2 knockout mice

GSE151571, containing heart specimens isolated from Mdm2 knockout mice, was enrolled as our in-vivo experimental cohort [27]. Using the R packages “GSVA” (version 1.48.0) [30] and “clusterProfiler” (version 4.8.1) [36], we employed the GSEA algorithm to investigate the enriched discrimination of four gene sets (p53 signaling, neutrophil apoptotic process and clearance, neutrophil, and neutrophil activation involved in immune response) between the control and Mdm2 knockout groups. In addition, ssGSEA was implemented to yield enrichment scores for the above-mentioned gene sets for each sample in the control and Mdm2 knockout groups. We then calculated the Pearson’s correlation coefficients between ssGSEA enrichment scores of different gene sets (p53 signaling *versus* neutrophil apoptotic process and clearance; p53 signaling versus neutrophil activation involved in immune response).

### Statistical analysis

Statistical analyses were conducted in R programming (version 4.3.0) with a two-sided P-value less than 0.05 regarded as statistically significant. Pearson’s correlation coefficients were utilized to evaluate the associations between two continuous variables. To compare the differences in continuous variables between the two groups, an unpaired Student’s t-test was applied after passing the normality test. Otherwise, the Wilcoxon rank sum test was conducted. Kruskal–Wallis’s test was used to compare the differences in continuous variables between more than two groups. For the evaluation index of prediction on binary categorical variables, the AUC score was calculated via receiver operating characteristic curve (ROC) analysis implemented in the R package “pROC” (version 1.18.2) [65]. Moreover, the confusion matrix was generated through the R package “caret” (version 6.0-94), which was further utilized to calculate accuracy, sensitivity, specificity, and F1 score.

## Results

### Identification of 25 pivotal neutrophil-related PCD genes via comprehensive screening

**Fig. 2:**
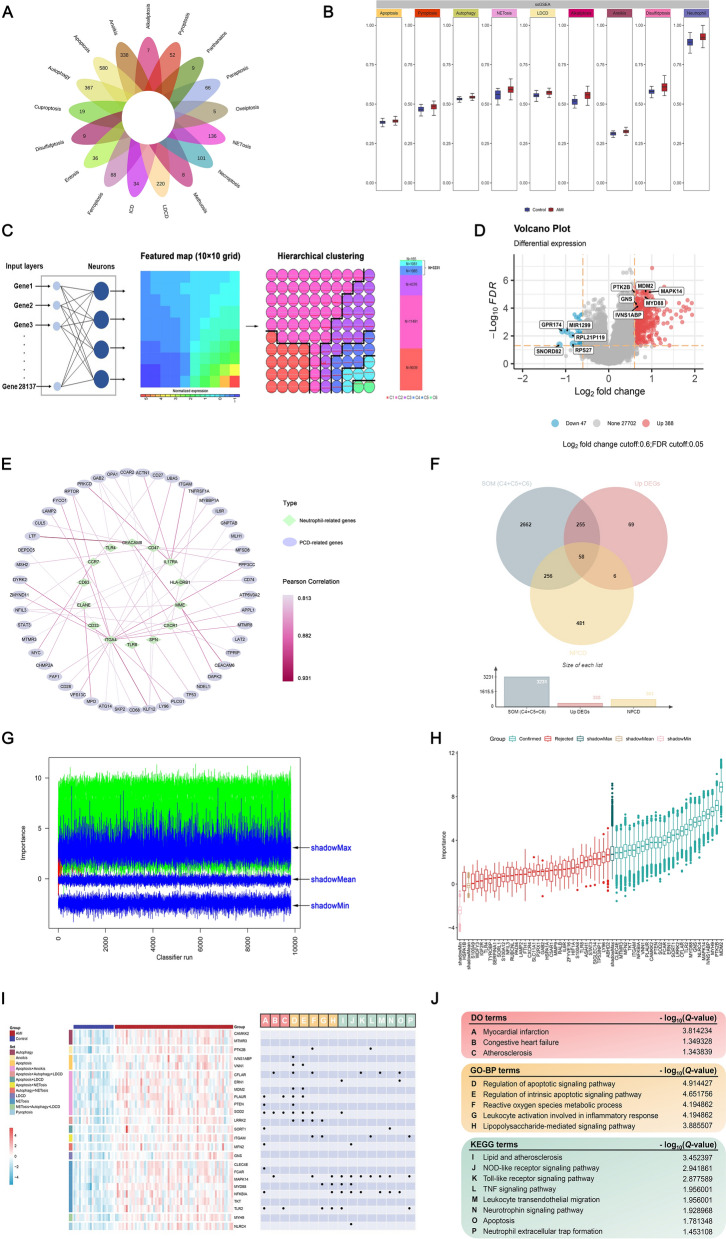
Identification of neutrophil-related PCD genes for NPCDS construction. **A** A collection of 1554 genes representing 17 PCD patterns. **B** The ssGSEA scores of 8 PCD patterns-related and neutrophil-related gene sets were significantly elevated in AMI compared with the control cases. **C** SOM clustering of the expression profile of AMI, and clusters 4–6 were defined as elevated gene signatures in AMI. **D** Linear model-based screening of DEGs between AMI and control cases. **E** The top 50 PCD-related genes were positively correlated to the expressions of neutrophil-related genes. **F** SOM, DEGs, and the correlation network co-identified 58 neutrophil-related PCD genes. **G** The iteration progress of the Boruta algorithm was performed on the expression profile of 58 neutrophil-related PCD genes. **H** The importance of 58 neutrophil-related PCD genes via the Boruta algorithm. A total of 25 neutrophil-related PCD genes were selected, which showed greater importance than the maximum shadow attribute. **I** The expression and PCD patterns of 25 neutrophil-related PCD genes. Left panel: Heatmap of 25 neutrophil-related PCD genes in AMI and control cases. Right panel: An enriched group of 25 neutrophil-related PCD genes. **J** DO, GO, and KEGG enrichment terms of 25 neutrophil-related PCD genes

We initially collected 1554 genes encompassing 17 PCD patterns, as displayed in Fig. 2A. Subsequently, we performed ssGSEA on the expression profile of GSE123342 leveraging 1554 PCD-associated genes (Supplementary Table 3). In comparison to control cases, we observed that the ssGSEA scores of 8 PCD patterns were significantly elevated in AMI (all P-value < 0.05), which comprised apoptosis, pyroptosis, autophagy, NETosis, LDCD, alkaliptosis, anoikis, and disulfidptosis (Fig. 2B). The above-mentioned PCD patterns were included in the latter analysis. Additionally, an up-regulated ssGSEA score of neutrophil-related genes was observed in AMI (P-value < 0.05). Next, we employed SOM clustering on the expression profile of AMI cases, with a featured map (10 × 10 grid) as input space (Fig. 2C). Coupled with hierarchical clustering, SOM could partition gene expression patterns into neighboring clusters in the featured map [66]. A total of six clusters were identified (Fig. 2C), whereas three clusters (C4, C5, and C6) were defined as AMI-sensitized expression patterns. Genes in each cluster can be found in Supplementary Table 4. As depicted in Fig. 2D, we screened 435 DEGs between AMI and control cases, including 388 up-regulated and 47 down-regulated genes. The details of DEGs are summarized in Supplementary Table 5. To gain insight into genes involved in PCD-neutrophil crosstalk, we calculated the Pearson’s correlation coefficients between the expressions of 8 PCD patterns and neutrophil-related genes (Supplementary Table 6). We obtained 801 neutrophil-related PCD genes (R > 0.6 and P-value < 0.001), and the top 50 PCD genes strongly interacting with neutrophil genes were demonstrated in Fig. 2E.

Taking the intersection between 3231 genes from three SOM-identified clusters, 388 up-regulated genes in AMI, and 801 neutrophil-related PCD genes, we gathered 58 overlapping genes (Fig. 2F). The Boruta algorithm was further adopted to narrow down the intersected 58 genes to 25 genes confirmed to be more relevant to AMI. As presented in Fig. 2G, Boruta was executed in 9828 iterations until all genes were captured with a decision (confirmed or rejected). After comparing the importance of each feature and shadow attribute, we ranked the importance of each gene. We retained 25 genes displayed with a more significant degree than the maximum shadow attribute (Fig. 2H). Afterward, we demonstrated the expression and PCD patterns of Boruta-selected 25 genes in control and AMI cases. As shown in Fig. 2I, we observed that the 25 genes were up-regulated in AMI and belonged to diverse PCD patterns (apoptosis, anoikis, autophagy, NETosis, LDCD, and pyroptosis). Then, we performed DO, GO, and KEGG enrichment analyses on these 25 genes, and significantly enriched terms are shown in Fig. 2I and J. Regarding DO terms, the top 3 were myocardial infarction, congestive heart failure, and atherosclerosis. The main BP terms of DO analysis were regulation of apoptotic signaling pathway, reactive oxygen species metabolic process, and leukocyte activation involved in inflammatory response. Additionally, we found that the enriched KEGG terms were lipid and atherosclerosis, Toll-like receptor signaling pathway, TNF signaling pathway, leukocyte transendothelial migration, neurotrophin signaling pathway, apoptosis, and neutrophil extracellular trap formation. These results indicated that the 25 neutrophil-related PCD genes were closely relevant to biological processes or pathways involved in AMI.

### Neutrophil-related PCD signature (NPCDS) generated from an integrated ML-based framework

**Fig. 3:**
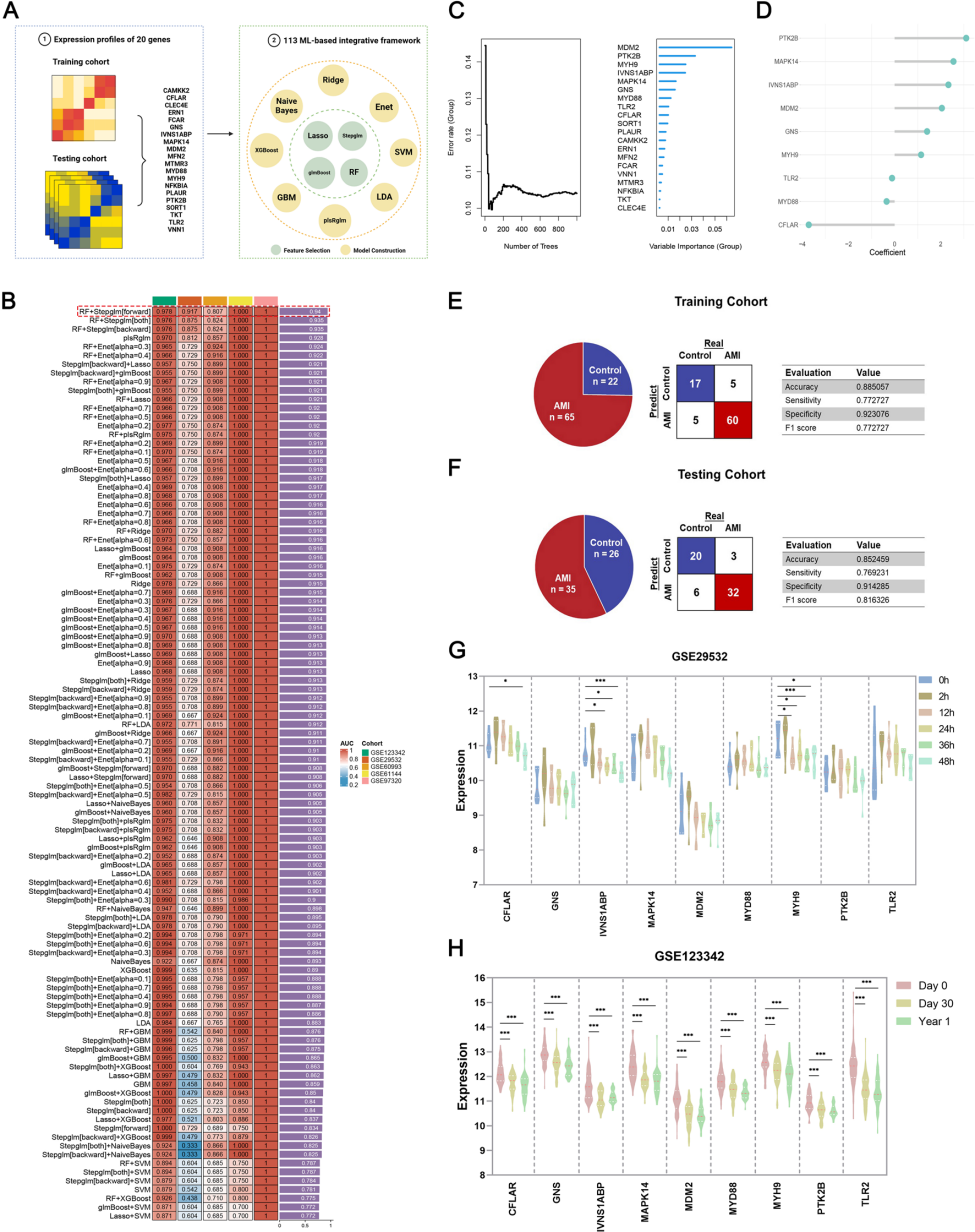
An integrated ML-based framework developed a neutrophil-related PCD signature (NPCDS) of diagnostic significance. **A** A total of 20 neutrophil-related PCD genes were subjected to the ML-based framework. **B** AUC scores were generated via 113 prediction models in training and testing cohorts. **C** RF algorithm selected 9 feature genes from 20 neutrophil-related PCD genes. **D** The coefficient profiles of 9 feature genes were obtained through the stepwise GLM algorithm. **E** Overview of control and AMI samples utilized in the training cohort and the NPCDS-derived confusion matrix. **F** Overview of the control and AMI samples utilized in the testing cohort and the NPCDS-derived confusion matrix. **G** The expression patterns of 9 feature genes in AMI patients before treatment (0 h) and after treatment within 12 h, 24 h, 36 h, and 48 h. **H** The expression patterns of 9 feature genes in first-discovery AMI (Day 0), 30 days post-AMI (Day 30), and 1-year post-AMI (Year 1). Kruskal–Wallis’s test was performed; ^*^P-value < 0.05, ^**^P-value < 0.01, ^***^P-value < 0.001 (Fig. 3A was created with BioRender.com with premission)

By overlapping the genes in five independent cohorts (GSE123342, GSE29532, GSE60993, GSE61144, GSE97320), we identified 20 neutrophil-related PCD genes for NPCDS construction (Fig. 3A). GSE123342 was selected as the training cohort for discovering NPCDS, while GSE29532, GSE60993, GSE61144, and GSE97320 were the independent testing cohorts (Fig. 3A). Subsequently, the expression profiles of these 20 neutrophil-related PCD genes were subjected to an integrated ML-based framework to develop NPCDS. We employed 113 model combinations using twelve ML algorithms in this framework, whereas the combination with the highest average AUC in five cohorts was considered optimal. As depicted in Fig. 3B, the best-performing model was a combination of RF and Stepglm (forward), with a leading mean AUC score (0.94) in five cohorts. We called this combination of RF and Stepglm (forward) NPCDS. Notably, the AUC scores of NPCDS performed in all five cohorts were greater than 0.8, suggesting the NPCDS’s robust generalization ability. The progress of NPCDS generation was shown in Figs. 3C and D. In the RF algorithm, the top 9 important genes (MDM2, PTK2B, MYH9, IVNS1ABP, MAPK14, GNS, MYD88, TLR2, CFLAR) were selected from the 20 input genes under a tenfold CV (Fig. 3C). These nine RF-selected genes were then subjected to the Stepglm algorithm to fit the regression model, and the expression of each gene was weighted via its corresponding regression coefficient (Fig. 3D). Leveraging NPCDS to yield prediction probabilities and predicted labels for each case, confusion matrices were generated for the training cohort (Fig. 3E) and testing cohort (Fig. 3F). Apart from the AUC score, the other four bicategory indicators for assessing NPCDS were calculated, including accuracy, sensitivity, specificity, and F1 score. Regarding the balanced and imbalanced class distributions in our enrolled cohorts, the interrogation of high accuracy (> 0.85), sensitivity (> 0.75), specificity (> 0.9), and F1 score (> 0.75) again underscored the accurate and reliable diagnostic performance of NPCDS to distinguish AMI from control cases. Furthermore, to validate our NPCDS more rigorously, we measured its prediction ability in the external validation cohorts. The robust predictive performance of NPCDS was again demonstrated in GSE48060 (AUC = 0.9, Supplementary Fig. 2A) and GSE194388 (AUC = 0.94, Supplementary Fig. 2B). To gain more insight into the dynamics of NPCDS expressed across AMI progression, we investigated the expression patterns of NPCDS in AMI cases collected at different time points in GSE29532 and GSE123342. As illustrated in Fig. 3G, the expressions of CFLAR, MYH9, and IVNS1ABP were found to decrease in AMI after mechanical reperfusion and stenting. Interestingly, compared with patients who were first diagnosed with AMI, the downregulated expression patterns of all nine genes were observed in cases of 30 days post-AMI and one-year post-AMI (Fig. 3H). This evidence suggested the crucial role of NPCDS in the early phase of AMI, which also emphasized its underlying application in early prediction and intervention.

### Comparison of NPCDS and other published signatures

**Fig. 4:**
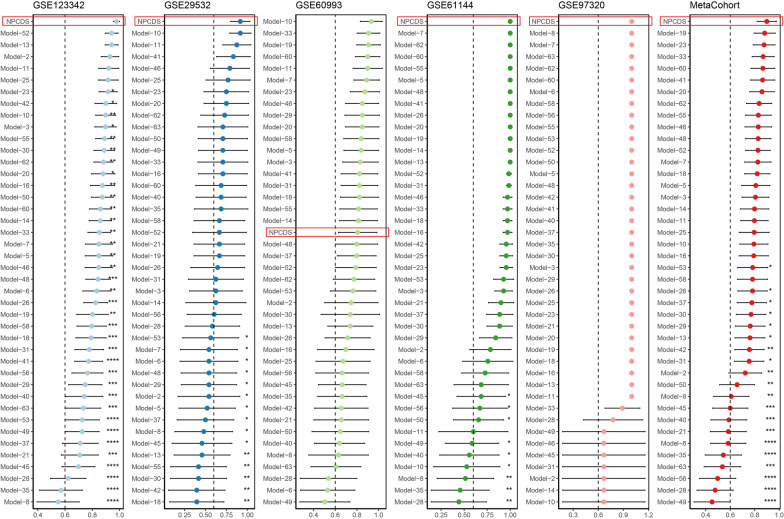
Comparison of NPCDS and published gene expression signatures. For the predictive performance of each signature, AUC scores were calculated in five cohorts and the meta-cohort. An unpaired student’s test was performed; ^*^P-value < 0.05, ^**^P-value < 0.01, ^***^P-value < 0.001

With the advent of sequencing techniques and computational approaches, masses of gene expression signatures have been developed for disease prediction. To ensure comparability between NPCDS and other signatures, we curated the mRNA expression signatures utilized in AMI prediction. Ultimately, we enrolled 63 published signatures (Supplementary Table 7). Of note, some of these signatures were derived from diverse functional gene sets, such as immune cell characteristics, extracellular matrix, ferroptosis, senescence, and cuproptosis. We filtered out signatures with more than 30% of mismatched genes in the five independent cohorts, and a final set of 41 signatures was retained. For each signature for comparison, the AUC score was estimated in all cohorts and the meta-cohort. Next, we compared the AUC score of NPCDS with other enrolled signatures. As illustrated in Fig. 4, the AUC performance of NPCDS ranked first in GSE123342, GSE29532, GSE61144, GSE97320, and Meta-cohort, whereas it ranked nineteenth in GSE60993. Additionally, we noticed the robust predictive performance of our NPCDS across the two external validation cohorts (Supplementary Fig. 2C), ranking first in GSE48060 and second in GSE194388. Thereby, our NPCDS model demonstrated better diagnostic efficacy than almost all collected signatures (P-value < 0.05) in cohorts apart from GSE60993. We noticed that the first-ranked AUC score was signature 10, which was a ten-gene panel composed of TNF, CXCL8, FPR2, CXCL1, JUN, IL1B, PPBP, MMP9, FCER1G, and LILRB2 [67]. Nevertheless, signature 10 performed weakly in other cohorts, particularly GSE61144 (ranking 39 of 42) and GSE97320 (ranking 42 of 42), suggesting its poor generalization ability brought on by overfitting. Similarly, this issue also existed for the other signatures ranked before NPCDS in GSE60993. Additionally, NPCDS remained the best-performing one within the meta-cohort that integrates all cohorts. Hence, our NPCDS model was again demonstrated to be capable of precisely predicting AMI, which also exhibited a more robust generalization ability than other published signatures.

### Robust prediction performance of NPCDS and development of a nomogram incorporating clinical characteristics

**Fig. 5:**
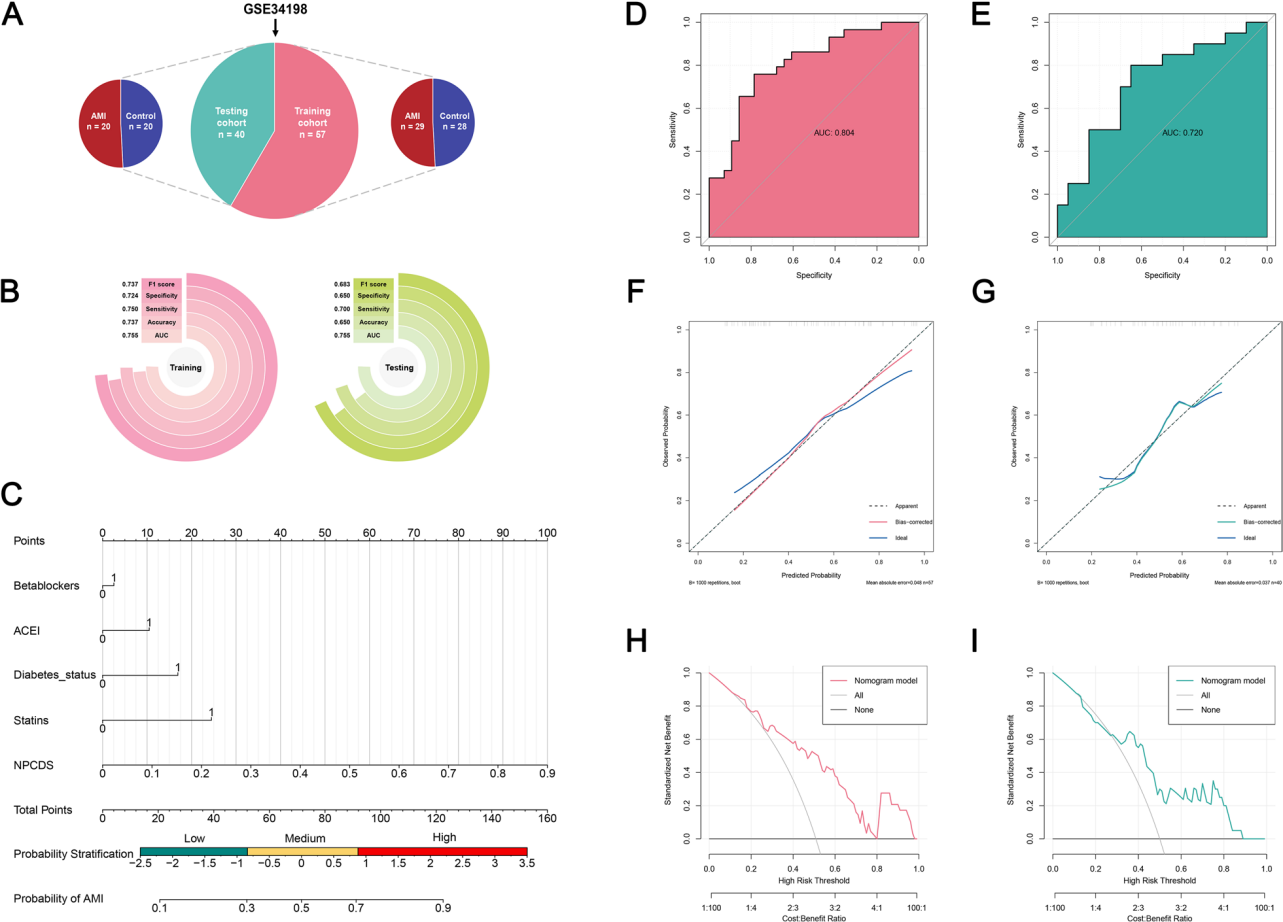
External validation of NPCDS and the development of a nomogram incorporating clinical information. **A** The external clinical dataset (GSE34198) was classified into the GSE34198-training and GSE34198-testing cohorts in a ratio of 6:4. **B** Evaluating the predictive performance of NPCDS in the GSE34198-training and GSE34198-testing cohorts. **C** NPCDS and clinical information were incorporated into developing a nomogram. ROC analysis of the nomogram in the GSE34198-training cohort (**D**) and GSE34198-testing cohort (**E**). Calibration curve of the nomogram in the GSE34198-training cohort (**F**) and GSE34198-training cohort (**G**). DCA curve of the nomogram in the GSE34198-training cohort (**H**) and GSE34198-testing cohort (**I**)

External validation for evaluating NPCDS was also implemented in the GSE34198 cohort, which contained 48 control and 49 AMI cases. Next, we randomly separated GSE34198 into the GSE34198-training cohort (29 control and 28 AMI cases) and the GSE34198-testing cohort (20 control and 20 AMI cases) according to a ratio of 6:4 (Fig. 5A). The robust predictive performance of NPCDS within GSE34198 (Fig. 5B) was again benchmarked by multifold indicators encompassing AUC (all > 0.75), accuracy (all > 0.65), sensitivity (all > 0.7), specificity (all > 0.65), and F1 score (all > 0.65). Regarding the clinical generalization and model optimization of NPCDS, a logistic regression-based nomogram incorporating AMI-relevant clinical characteristics and NPCDS was further constructed. After employing logistic regression, we obtained four significant clinical characteristics (betablockers, ACEI, diabetes status, stains) and NPCDS to develop a nomogram (Fig. 5C). The uppermost scale of the nomogram represents the point assignment for each variable. And the second to fifth scales stand for the nomogram-enrolled variables, namely betablockers, ACEI, diabetes status, stains, and NPCDS. By establishing a vertical line between the variable and point scales, each variable will be given a point value on the point scale. For each individual, the nomogram could sum a total point (seventh scale) by adding up the assigned points of all variables. Once the total point was obtained, the probability stratification (eighth scale) and probability of AMI (lowermost scale) could be predicted through locating the point value on the total point scale. According to the range of prediction probability, we also defined an AMI stratification consisting of low-risk (0–0.3), medium-risk (0.3–0.7), and high-risk (0.7–1.0). To assess the comprehensive performance of the nomogram, we subsequently adopted three commonly-used indexes, including discrimination (AUC), calibration, and DCA. Remarkably, our nomogram showed better diagnostic ability than NPCDS in the GSE34198-training cohort (AUC = 0.804), as depicted in Fig. 5D. The AUC score of our nomogram within the GSE34198-testing cohort abated in comparison with standalone usage of NPCDS (AUC = 0.720, Fig. 5E). The calibration curves demonstrated that the nomogram-predicted probabilities were approximately consistent with the actual probabilities within the GSE34198-training cohort (Fig. 5F) and GSE34198-testing cohort (Fig. 5G), which indicated a better performance with minor predictive deviation. The clinical practicability of our nomogram was assessed by the DCA curves, as depicted in Fig. 5H and I. The net benefits of the nomogram were greater than those of the “none” and “all” baselines under the high-risk threshold ranging from 0.4 to 1.0. This result suggested that the nomogram could contribute to clinical decision-making for AMI patients predicted with medium-risk (0.3–0.7) and high-risk (0.7–1.0). Overall, our nomogram integrating clinical characteristics and NPCDS performed robustly in predicting AMI, especially those stratified as medium or high risk.

### Two molecular subtypes were established by leveraging NMF clustering on NPCDS

**Fig. 6:**
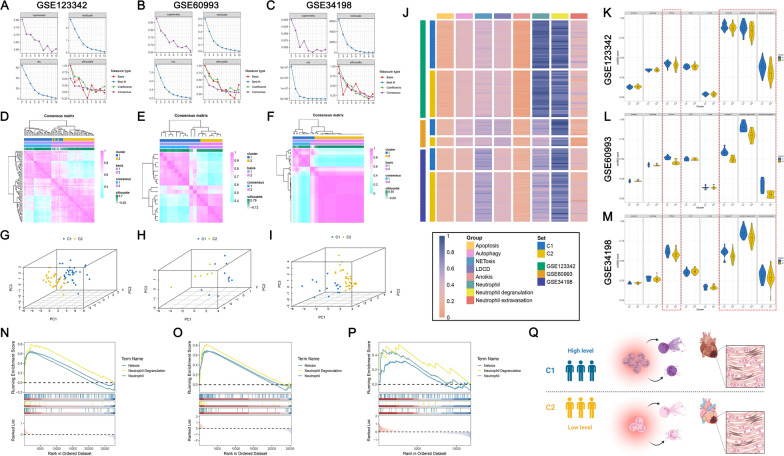
Construction of molecular subtypes of AMI leveraging the NMF algorithm on NPCDS. Evaluating the NMF indicators of cophenetic, residuals, RSS, and silhouette with ranks ranging from 2 to 10 in GSE123342 (**A**), GSE60993 (**B**), and GSE34198 (**C**). Consensus heatmaps demonstrating the AMI patients from GSE123342 (**D**), GSE60993 (**E**), and GSE34198 (**F**) were classified into two clusters when rank equals two. The two-dimensional PCA score plots showed the distribution of two clusters in GSE123342 (**G**), GSE60993 (**H**), and GSE34198 (**I**). **J** The activity patterns of 8 gene sets in two clusters from GSE123342, GSE60993, and GSE34198. The gene sets of NETosis, neutrophil, neutrophil degranulation, and neutrophil extravasation were significantly up-regulated in cluster 1 from GSE123342 (**K**), GSE60993 (**L**), and GSE34198 (**M**). GSEA on cluster 1 and cluster 2 from GSE123342 (**N**), GSE60993 (**O**), and GSE34198 (**P**) based on gene sets of NETosis, neutrophil, and neutrophil degranulation. **Q** Schematic illustration of differences in biological processes of neutrophils between cluster 1 and cluster 2. Kruskal–Wallis’s test was performed; ^*^P-value < 0.05, ^**^P-value < 0.01, ^***^P-value < 0.001 (Fig. 6Q was created with BioRender.com with premission)

At present, the subtypes of AMI are clinically widely determined according to their electrocardiography patterns, mainly encompassing ST-elevation MI and non-ST-elevation [68]. Nevertheless, investigations on molecular subtypes of AMI remain insufficient. Therefore, partitioning of AMI patients was employed using NMF clustering on NPCDS expressions. To guarantee the stability of implementing clustering, we enrolled the GSE123342, GSE60993, and GSE34198 datasets, all of which containing more than 15 AMI cases. Following the index criteria of NMF (cophenetic, residuals, RSS, and silhouette), we have determined the optimal value of rank value (k = 2) at which the magnitude of cophenetic begins to drop (Fig. 6A–C). Accordingly, we identified two molecular subtypes of AMI leveraging the optimal rank. Foremost, consensus heatmaps were generated to demonstrate the stability of clustering when k equals 2 (Fig. 6D–F). Furthermore, three-dimensional PCA plots displayed the distinct distribution of NPCDS among the two identified subtypes (Fig. 6G–I). Regarding whether the NPCDS-derived subtypes differ in clinical characteristics of AMI patients, we used the chi-square test to evaluate the differences between the two subtypes within the GSE34198 dataset. Clinical characteristics showed no significant differences among the two subtypes of GSE34198 (Supplementary Fig. 3). These results suggest that the partition of AMI patients can be better represented through the two subtypes.

We then implemented ssGSEA to measure the enrichment of signatures from NPCDS-affiliated PCD patterns (apoptosis, autography, NETosis, LDCD, anoikis) and neutrophils within two subtypes. We also evaluated the ssGSEA scores of the neutrophil processes well-known to be implicated in AMI dynamics, including neutrophil degranulation and neutrophil extravasation [17]. As depicted in Fig. 6J, the overall high-enriched pattern of NETosis, LDCD, neutrophil, and neutrophil degranulation was observed in two subtypes. Of note, we found that, compared with subtype 2, signatures from NETosis, neutrophil, neutrophil degranulation, and neutrophil extravasation in subtype 1 were significantly abundant (Fig. 6K–M). Next, GSEA was leveraged to assess whether NETosis, neutrophil, neutrophil degranulation, and neutrophil extravasation were relevant with subtype distinction (subtype 1 versus subtype 2; Supplementary Table 10). Figure 6N–P shows a positive correlation tendency of NETosis, neutrophil, and neutrophil degranulation significantly reached in subtype 1 (NES > 1 and FDR < 0.05). These activated neutrophil functions usually indicate a poorer clinical outcome for AMI. Thus, we speculated that AMI patients within subtype 1 demonstrated high infiltration levels of neutrophils, coupled with vibrant activities of NETosis and neutrophil degranulation (Fig. 6Q). Our results are potentially in support of a novel stratification of AMI patients on the basis of NPCDS.

### ScRNA-seq analysis uncovered the diverse dynamic patterns of NPCDS across neutrophil transformation

**Fig. 7:**
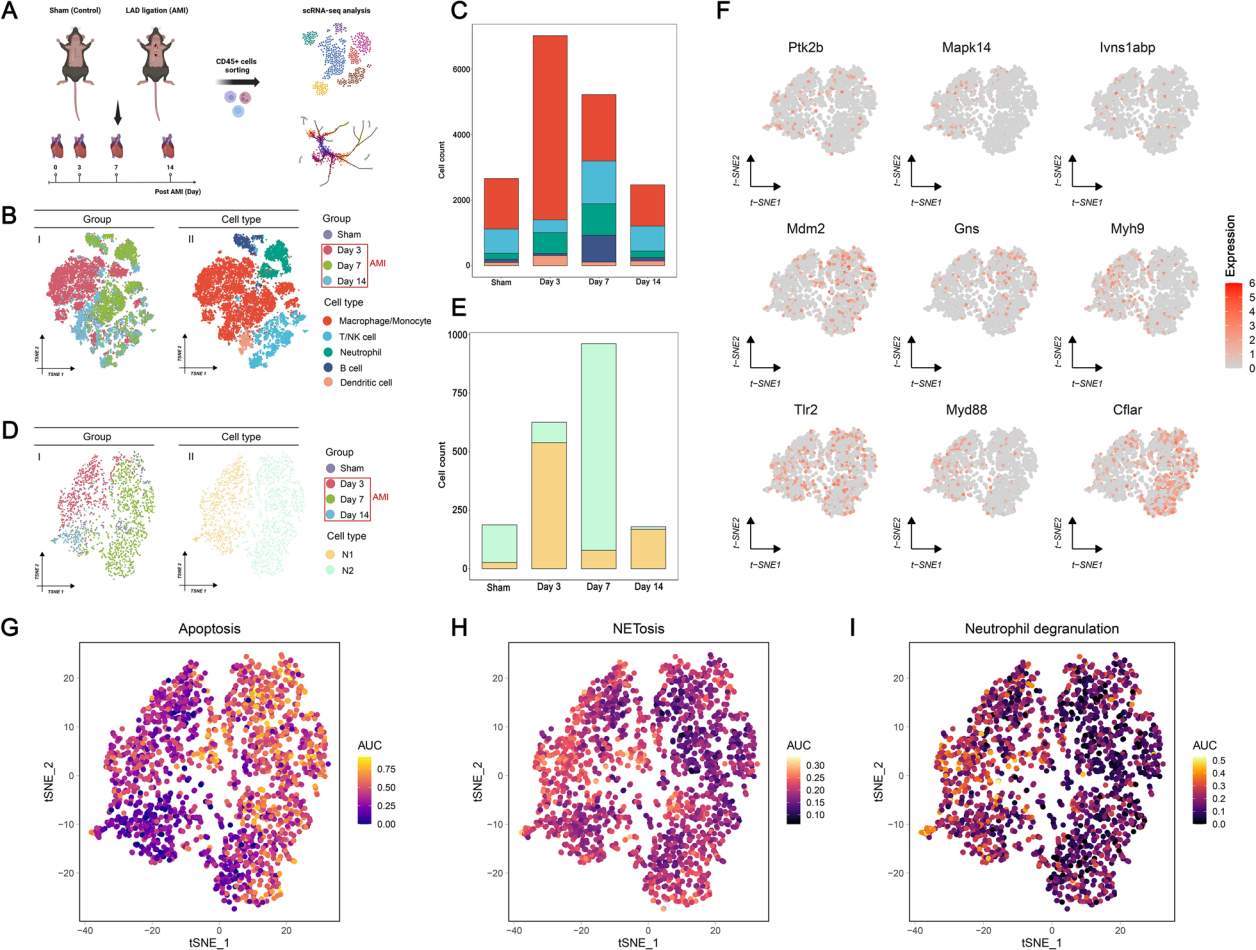
Investigating the expression pattern of NPCDS at single-cell resolution in the progression of AMI. **A** Schematic diagram of scRNA-seq analysis on Cd45 + cells isolated from hearts of control and AMI groups. **B** tSNE visualization of immune cell populations with type annotation (macrophage/monocyte, T/NK cell, neutrophil, B cell, and dendritic cell). **C** The numbers of each cell population in the control group (Sham) and AMI at different time points (Day 3, Day 7, Day 14). **D** tSNE visualization of the neutrophil population with subtype annotations (N1 and N2). **E** The numbers of each neutrophil subtype in the control group (Sham operation) and AMI at different time points (Day 3, Day 7, Day 14). **F** Expression distributions of feature genes in the N1 and N2 subtypes. The activity scores of feature genes (**G**), NETosis (**H**), and neutrophil degranulation (**I**) in the N1 and N2 subtypes (Fig. 7A was created with BioRender.com with premission)

To delve deeper into the mechanism of NPCDS in AMI progression, we implemented scRNA-seq analysis to illustrate the dynamics of NPCDS in the neutrophil transition. The GSE163465 dataset was recruited for our analysis, which contains Cd45+ cells sorted from murine hearts at different time points post-AMI (0, 3, 7, 14 days after LAD ligation modeling). The overall analysis flow is described in Fig. 7A. After employing quality trimming and filtering (Supplementary Fig. 4), we gathered a total of high-quality 17, 384 cells that highly expressed Cd45. In combination with unsupervised clustering and t-SNE reduction, we identified a final set of six clusters on the Cd45+ cells. Subsequently, well-established marker genes of cardiac immune cells were leveraged to annotate these six putative clusters (Supplementary Fig. 5). Single-cell profiling of cardiac Cd45+ cells was depicted in Fig. 7B, including macrophage/monocyte, T/NK cell, neutrophil, B cell, and dendritic cell. Figure 7C further demonstrated the dynamic alteration in the proportion of each cell population during the progression of AMI. Noteworthy is that we observed a substantial increase of neutrophils from day 3 to day 7 post-AMI, whereas there was a distinct decrease on day 14 after AMI. This result agrees with prior investigations describing changes in neutrophils infiltrated in AMI [17].

To gain more insight into the heterogeneity of neutrophils in AMI, we defined two distinct subpopulations of neutrophils (N1 and N2) using pro-inflammatory and anti-inflammatory marker genes (Supplementary Fig. 6, Fig. 7D). As shown in Fig. 7E, we noticed the opposite change pattern of N1 and N2 during AMI progression. The N1 subtype, which was reflected as high expressions of inflammatory markers (Ccl2, Ccl7, Ccl9, Isg15, Ifitm6, and Mmp8), increased sharply on day 3 after AMI and then significantly fell thereafter. Whereas the N2 subtype, which showed high expressions of apoptotic and anti-inflammatory markers (Sod2, Mif, Bnip3, Lgals3, Vim, and Il10), infiltrated in a small amount on day 3 post-AMI but rallied on day 7. Intriguingly, we detected two neutrophil subpopulations with opposite biological functions, namely N1 for inflammation and N2 for anti-inflammation. Moreover, we investigated the expression distribution of NPCDS in these two subpopulations. The N1 subtype exhibited significant expressions of Myd88 and Myh9, whereas the N2 subtype displayed strong upregulation of Cflar and Mdm2 (Fig. 7F). Leveraging the AUCell algorithm, we evaluated whether the AUCell scores calculated from NPCDS, NETosis, and neutrophil degranulation were significantly enriched within the neutrophil subtypes. We observed that signatures from apoptosis (Fig. 7G) were significantly enriched in N2, but NETosis (Fig. 7H) and neutrophil degranulation (Fig. 7I) were significantly enriched in N1. This result demonstrates that the N1 subtype corresponds to an active inflammatory phase accompanied by greater enrichment of NETosis and neutrophil degranulation, whereas the N2 subtype represents an anti-inflammatory phase.

**Fig. 8:**
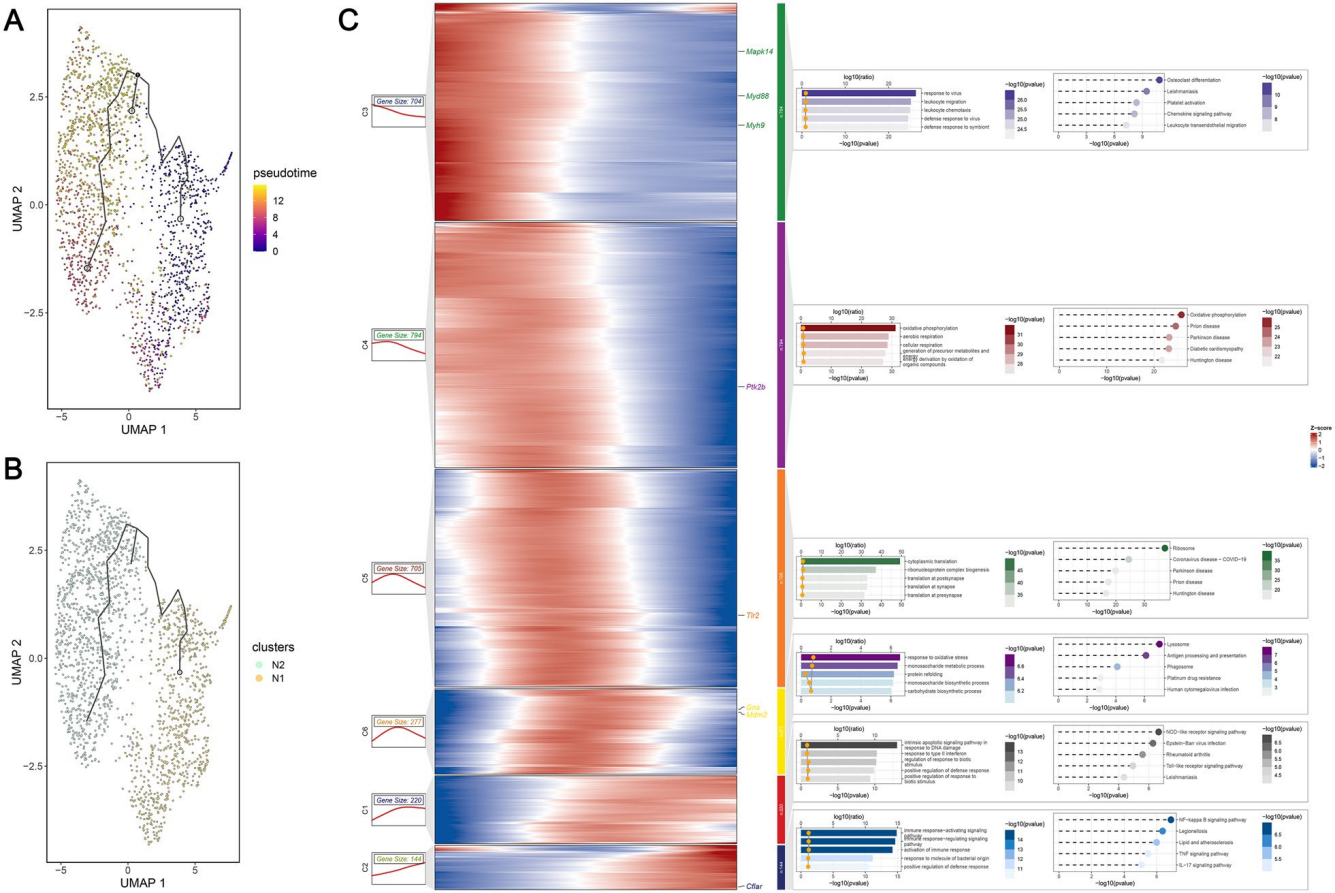
The diversity and dynamics of neutrophils in AMI progression uncovered the expression kinetics of NPCDS in the neutrophil transition. **A**, **B** Pseudotime analysis uncovered the transition from N1 to N2 subtypes. **C** Heatmap of pseudotime-ordered DEGs between N1 and N2 subtypes. Left panel: K-means clustering suggested six kinds of kinetics of DEGs expressions. Right panel: The top 5 GO and KEGG enrichment terms of each expression kinetics

Then, we implemented pseudotime analysis to infer the differential trajectory of neutrophils. As expected, N2 mainly appeared at the end of the trajectory with the higher putative pseudotime (Fig. 8A, B), indicating the transition from N1 to N2. As the expression pattern of NPCDS was previously proven to not be significantly dispersed in N1 and N2, we explored whether NPCDS undergoes dynamic changes during the neutrophil transformation. As depicted in Fig. 8C (left), we gathered six kinetic patterns (denoted by C1, C2, C3, C4, C5, C6) through clustering on genes that were differentially expressed along the trajectory. Regarding the dynamics of NPCDS along pseudotime, we noted that Mapk14, Myd88, Myh9, Ptk2b, and Tlr2 were allocated to a similar directional panel displaying expressions initially along pseudotime (C3, C4, C5), suggesting their premature activations in the N1 subtype with lower pseudotime. However, increasingly progressive changes of Gns, Mdm2, and Cflar were found across the pseudotime (C1, C2, C6), indicating the delayed activations in the N2 subtype with higher pseudotime. Next, we conducted GO and KEGG enrichment analyses to reveal the molecular programs illuminating the transformation from N1 to N2 (Fig. 8C, right). Notably, C3, C4, and C5 patterns mainly participated in biological processes or pathways related to leukocyte activation, migration, oxidative phosphorylation, and protein translation. C1, C2, and C6 patterns were significantly enriched in apoptotic-relevant processes or pathways, such as lysosome, intrinsic apoptosis pathway, Toll-like receptor signaling pathway, and NF-kB signaling pathway. Altogether, these results suggest the N1 subtype, with strong pro-inflammatory activities initially abundant in the early stage of AMI, will afterwards convert into N2 via revitalization of apoptosis-related pathways in the terminal stage. Given the great potential of apoptotic neutrophils to limit post-infarction inflammation, we hypothesized that the N1 to N2 transition could represent a switch from an inflammatory phase to a reparative phase during AMI, in which NPCDS dynamically regulates at these stages.

### Network inference unveiled the biological mechanisms underlying NPCDS in AMI

To decipher the biological mechanism underlying NPCDS in AMI, we first retrieved NPCDS-dominated signaling pathways from the KEGG database. We obtained a total of six pathway terms, including neutrophil extracellular trap formation (involvement of TLR2 and MAPK14; Supplementary Fig. 8A), Toll-like receptor signaling pathway (involvement of TLR2, MYD88, and MAPK14; Supplementary Fig. 8B), p53 signaling pathway (involvement of MDM2; Supplementary Fig. 8C), apoptosis (involvement of CFLAR; Supplementary Fig. 8D), lysosome (involvement of GNS; Supplementary Fig. 8E), and leukocyte transendothelial migration (involvement of PTK2B and MYH9; Supplementary Fig. 8F). IVNS1ABP is not recorded in the KEGG database but reportedly contributes to the dynamic stabilization of the actin cytoskeleton [69].

**Fig. 9:**
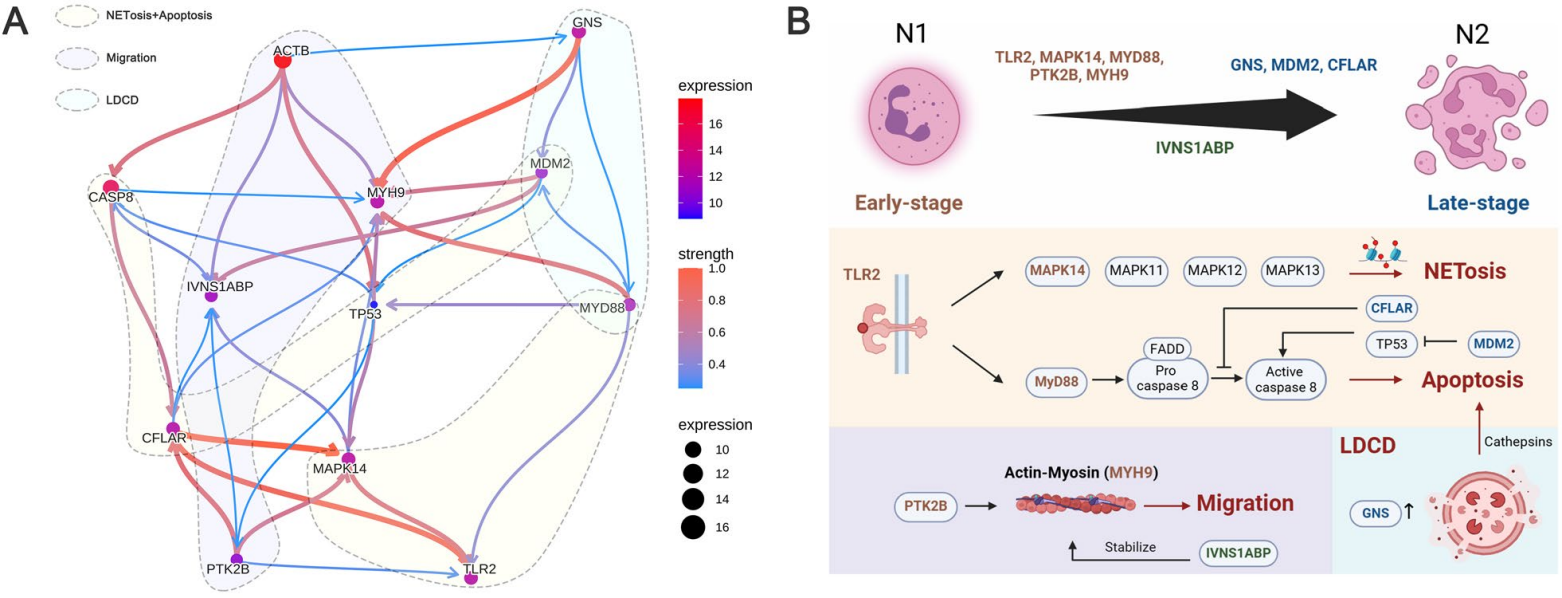
A combination of the KEGG database and Bayesian inference revealed the NPCDS-dominated molecular characteristics in AMI. **A** The Bayesian-inferred network between NPCDS and their regulatory genes was based on the KEGG retrieval pathway. **B** Biological interpretation of the molecular characteristics of NPCDS during the neutrophil transition in AMI (Fig. 9B was created with BioRender.com with premission)

We then leveraged NPCDS and their interactive genes that participated in curated pathways to GRN construction. As displayed in Fig. 9A, we explicitly inferred a GRN incorporating NPCDS and the interactive genes after implementing Bayesian network analysis. Intriguingly, we found that TLR2 interacts with MAPK14 and MYD88 with high confidence, which is directionally consistent with the known interplay in NETosis and apoptosis. With regard to MDM2 and CFLAR involved in apoptosis, we noted close directional linkages between MDM2, CFLAR, TP53, and CASP8, in which MDM2 regulates TP53, which in turn acts on CASP8. The interactions between CFLAR and CASP8 were observed. Moreover, we depicted the modulation of PTK2B and IVNS1ABP on the stabilization of MYH9 binding to ACTB, thereby contributing to neutrophil migration. Notably, we observed the regulatory effect of GNS on MDM2 and MYD88, suggesting the promotion of apoptosis via GNS upregulation, which broadened our knowledge of the potential function of GNS apart from the lysosome marker. More importantly, coupled with prior scRNA-seq analysis illuminating NPCDS dynamically expressed in neutrophil transformation, we speculated on the biological mechanism underlying NPCDS during the neutrophil transition in AMI (Fig. 9B). Strikingly, genes highly expressed in the early stage of the N1 subtype, including TLR2, MAPK14, MYD88, PTK2B, and MYH9, appeared upstream of their corresponding targeted pathways. We found that MDM2, CFALR, and GNS, which showed upregulations in the late stage of the N2 subtype, act as the downstream regulators in apoptosis and LDCD. Altogether, our results suggest that the pathways regulated via NPCDS, including NETosis, apoptosis, neutrophil migration, and LDCD, may represent the molecular program governing N1-N2 transformation in the progression of AMI.

### Causal effect and druggability of MDM2 on AMI

**Fig. 10:**
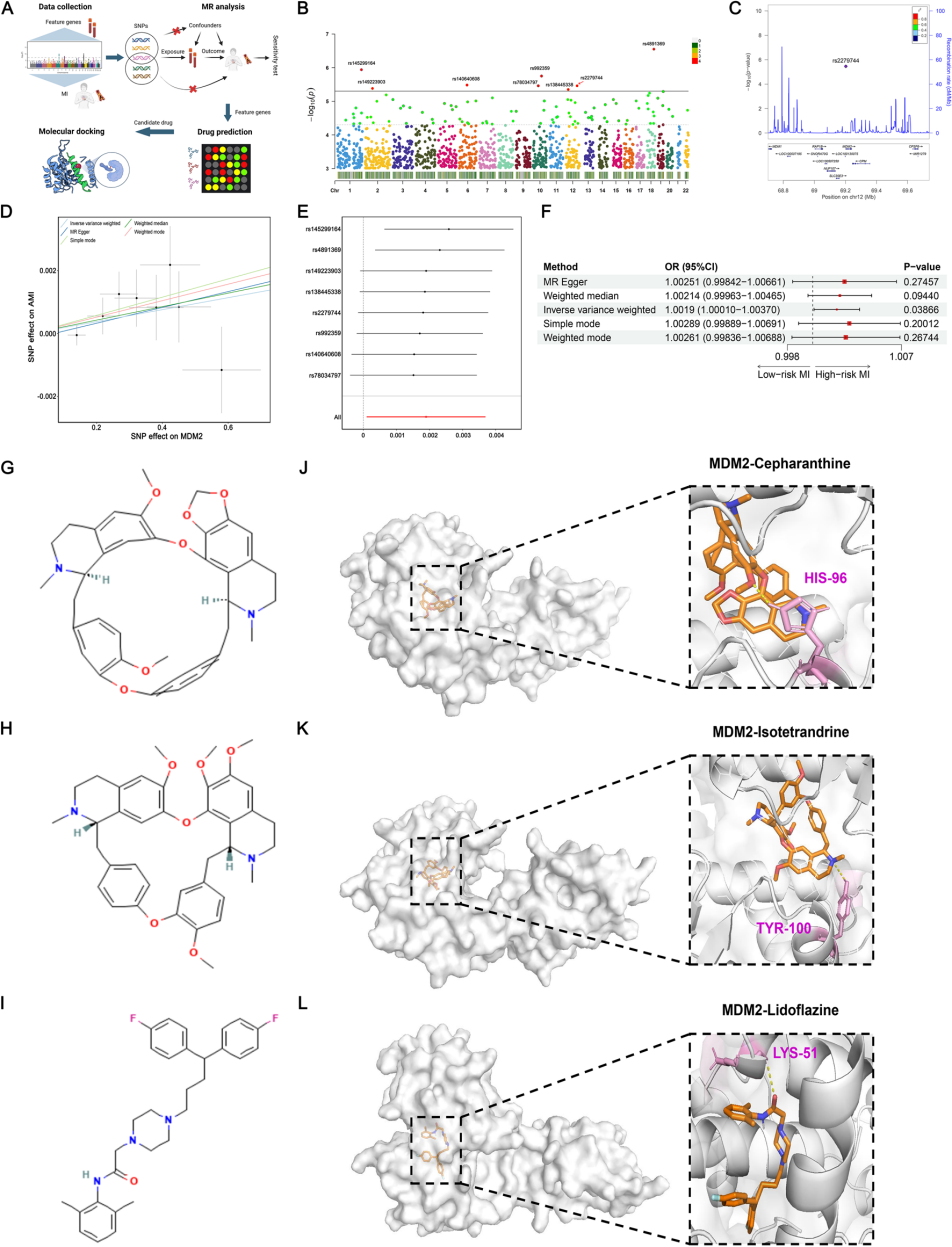
MDM2 was suggested as a prospective target of AMI through MR analysis and in-silicon docking. **A** A schematic overview of analytic flow combined with MR analysis and molecular docking on MDM2. **B** Manhattan plot of eight SNPs significantly associated with MDM2 (p < 5 × 10^–6^) selected as instrument variables of MR analysis. **C** Regional association plot of SNPs with MDM2 ± 500 kb from the locus on chromosome 12. rs2279744 (purple dot) represents the lead SNP highly associated with genetic liability to MDM2. **D** Scatterplot showing the positive association between the SNP effect on MDM2 (x-axis) and AMI (y-axis). **E** LOOCV plot demonstrating the SNP effect of MDM2 on AMI when removing one SNP step-by-step. **F** Forest plot displaying the estimated OR effects (95% confidence intervals) and P-values of MDM2 on the risk of AMI. The two-dimensional chemical structures of cepharanthine (**G**), isotetrandrine (**H**), and lidoflazine (**I**). Three-dimensional visualization of MDM2 docking cepharanthine (**J**), isotetrandrine (**K**), and lidoflazine (**L**) (Fig. 10A was created with BioRender.com with premission)

We sought to investigate the causal relationship and druggability of NPCDS on AMI. The overall analysis procedure is shown in Fig. 10A, and we reported results of MDM2 that demonstrated a significantly positive MR test. Leveraging two-sample MR analysis integrating exposure data (MDM2; ID: prot-a-1873) and outcome data (AMIID: ebi-a-GCST90038610), we assessed the causal relationship between the expression of MDM2 and the risk of AMI. Following three assumptions of the MR methodology, we selected a total of eight significant SNPs as strong IVs to conduct analysis (Fig. 10B). Among these selected SNPs, rs2279744 stands for the sentinel SNP in the MDM2 locus (± 500 kb), suggesting its close genetic liability to MDM2 expression (Fig. 10C). As depicted in Fig. 10D, we related the effect size of the SNP-MDM2 relationship (x-axis, SD) and the SNP-AMI relationship (y-axis, OR), and there was a positive slope intercept observed in all five MR methods. In a leave-one-out analysis, we sequentially excluded one SNP at a time to assess the sensitivity of result to each genetic variant, and no incorporated SNP was found to significantly influence the result (Fig. 10E). Sensitivity analysis further showed no heterogeneity and horizontal pleiotropy observed (all P-value > 0.05, Supplementary Table 14), guaranteeing the reliability of MR results. The IVW approach, with the most robust statistical power, indicated that the expression of MDM2 could increase the risk of AMI (OR > 1, P-value < 0.05; Fig. 10F). Though no significant statistical significance was generated from the other four MR methods, a positive association between MDM2 and AMI remained with consistent directional change (all OR > 1).

Given the great potential of MDM2 as a therapeutic target, a panel of small molecule drugs targeting MDM2 was initially predicted via the Enrichr database (Supplementary Table 15). Among the top five drugs prioritized by P-value and combined score, we initially excluded ruthenium and paclitaxel reportedly exhibiting cardiotoxicity. We retained a final set of three candidate drugs, including lidoflazine (Fig. 10G), isotetrandrine (Fig. 10H), and cepharanthine (Fig. 10I). To measure the affinity of drug-target (MDM2) interaction and from this to understand the druggability of MDM2, AutoVina was implemented to dock the three selected drugs (ligand) with MDM2 (receptor). Furthermore, we visualized the docking models with the lowest binding affinity and RMSE lower than 2 Å through PyMOL (Fig. 10J–L, left). The binding pocket of MDM2 could successfully encompass the three candidate drugs. Additionally, the formations of hydrogen bonds that drugs bind to the amino acids of MDM2 were analyzed (Fig. 10J–L, right), thus revealing the specific and stable binding patterns. Overall, our data suggest the positive causal effect of MDM2 on AMI risk and its underlying druggable value.

### In vivo experiments indicate loss of Mdm2 contributes to aggravated neutrophil apoptosis and inhibits neutrophil activation in mice

**Fig. 11:**
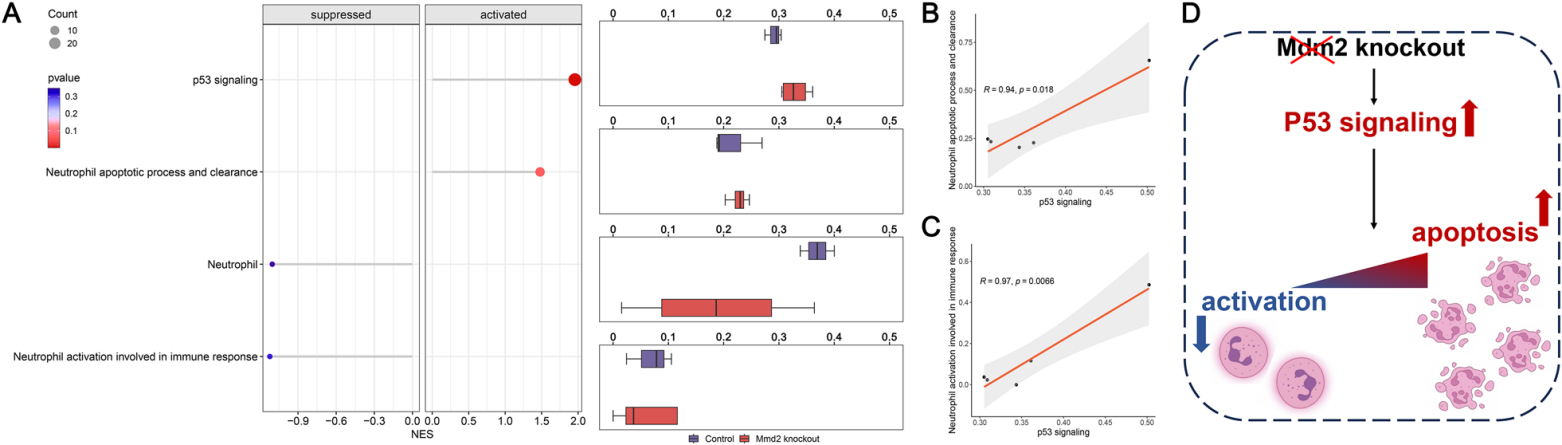
Knockout of Mdm2 aggravates neutrophil apoptosis and attenuates neutrophil activation in mice. **A** GSEA (left panel) and ssGSEA (right panel) analyses from four gene sets (p53 signaling, neutrophil, neutrophil apoptotic process and clearance, neutrophil, neutrophil activation involved in immune response) in the control and Mdm2 knockout groups. **B** Correlation of ssGSEA enrichment scores for p53 signaling and neutrophil apoptotic process and clearance in the Mdm2 knockout group. **C** Correlation of ssGSEA enrichment scores for p53 signaling and neutrophil activation involved in immune response in the Mdm2 knockout group. **D** Mechanism diagram illustrating Mmd2 knockout affecting the activities of neutrophils

To provide mechanistic insight into the molecular regulation of Mdm2 on neutrophil activities, we recruited GSE151571 (Mdm2 knockout *versus* control with Mdm2 expression) as our in-vivo experimental cohort [27]. We initially performed GSEA to examine the enrichment discrimination of four gene sets between the knockout and control groups, including p53 signaling, neutrophil apoptotic process and clearance, neutrophil, neutrophil activation involved in immune response. As depicted in Fig. 11A (left panel), GSEA revealed that p53 signaling and neutrophil apoptotic process and clearance were actively enriched in the Mdm2 knockout group (NES > 1). While neutrophil and neutrophil activation involved in immune response were suppressed (NES < 1). Furthermore, we implemented ssGSEA to quantify enrichment scores. As shown in Fig. 11A (right panel), the enrichment scores of p53 signaling and neutrophil apoptotic process and clearance in the knockout group demonstrated higher values compared to the control. Meanwhile, gene sets from neutrophil and neutrophil activation involved in immune responses presented lower enrichment scores. Additionally, we observed a significantly positive correlation between p53 signaling and neutrophil apoptotic process and clearance (Fig. 11B) as well as neutrophil activation involved in immune response (Fig. 11C), suggesting the close regulation of p53 signaling on neutrophil activities. Overall, these results indicate that loss of Mdm2 appeared to elevate activities of p53 signaling, which subsequently aggravated neutrophil apoptosis and attenuated neutrophil activation (Fig. 11D).

## Discussion

Despite the advancements in diagnosis and treatment, AMI remains high in both morbidity and mortality worldwide [70], posing a great threat to the medical burden. Within the first hours of the onset of AMI, the risk of mortality appears to be the highest. Hence, prompt detection of AMI is essential to improve this situation. Yet the currently available biomarkers, characterized by the most renowned cTn, are still far from the criteria of an ideal biomarker for AMI diagnosis. Owing to the boosted development of bioinformatics approaches, increasing evidence points to the rapid identification of novel disease biomarkers through omics information. However, the choice of analytic datasets largely depends on personal preference, which may hamper the reliability and applicability of identifications. Moreover, biomarkers generated via computational tactics generally lack interpretability, which limits our ability to explore them further. Current in-silicon studies involving AMI biomarkers suffer from these issues, and we attempt to overcome this limitation. An advantage of our work is that we meticulously curated single-source specimens, which are gene expression profiles from peripheral blood, to ensure diagnostic-specificity for AMI prediction.

Neutrophils, as the most abundant leukocyte in peripheral blood, are the first recruited to the infraction heart. Upon ephemeral activation and execution of the effector function, neutrophils initiate mechanisms of PCD [20, 71]. Given the key role of PCD in modulating neutrophil reactions post-AMI, we thereby hypothesized the great potential of neutrophil-derived PCD genes from peripheral blood in predicting and classifying AMI. We noted that eight divergent PCD patterns, including apoptosis, pyroptosis, autophagy, NETosis, LDCD, alkaliptosis, anoikis, and disulfidptosis, were significantly activated in AMI. Among these PCD patterns, previous reports have underscored the molecular programs of apoptosis, pyroptosis, autophagy, and NETosis in triggering neutrophil death [20, 71]. But information about investigations of LDCD, alkaliptosis, anoikis, and disulfidptosis on neutrophil death is relatively limited. We then developed a meta-analysis approach, which consists of SOM, DEGs, correlation, and Boruta analyses, to explore the crosstalk between the neutrophils and eight PCD patterns in AMI. We gathered a set of 25 neutrophil-derived PCD genes that firmly displayed an up-regulation pattern in AMI. With the expression profiles of these neutrophil-derived PCD genes, we leveraged an integrative ML-based computational framework to establish an NPCDS for predicting AMI. A total of 12 prevalent binary-classification ML algorithms were combined in our framework. In total, 113 ML combinations were benchmarked and tuned under the tenfold CV rule, thereby selecting more potent features and generating a more robust prediction model. Ultimately, the optimal model named NPCDS was determined by the combination of RF and Stepglm (forward) fitted on expressions of nine neutrophil-derived PCD genes (MDM2, PTK2B, MYH9, IVNS1ABP, MAPK14, GNS, MYD88, TLR2, CFLAR), which performed the highest average AUC score (0.94) within five independent cohorts. Notably, among these nine neutrophil-derived PCD genes to generate NPCDS, CFLAR demonstrated a significant negative coefficient (− 3.721) despite its up-regulation in AMI. This observation highlights the complexity of genetic contributions to AMI, which greatly reflects the intricacies of biological pathways that were adjusted for in the multivariable model [72]. And this reversal underscores the necessity of considering a broader gene-crosstalk to accurately elucidate gene-disease relationships. Hence, we sought to investigate the interplay of these nine neutrophil-derived PCD genes in the latter pathway analysis.

To subsequently investigate the generalizability and extensibility of NPCDS, we retrieved other published signatures for comparison and external independent cohorts for revalidation. In comparison of predictive superiorities among our 63 collected published gene-expression signatures, our NPCDS model was shown to precisely detect AMI with a better generalization capability, possessing the first-ranked AUC scores within almost all cohorts. Among these enrolled signatures of AMI prediction, some presented poor generalizability, and very few have been rigorously validated. For instance, we noticed that signature 10, a ten-gene panel composed of TNF, CXCL8, FPR2, CXCL1, JUN, IL1B, PPBP, MMP9, FCER1G, and LILRB2, performed marginally better than NPCDS in a specific cohort but was unsatisfactory across other cohorts. This undesirable generalizability may be attributed to overfitting using redundant genes with less biologically significant connections [73]. Notably, both the robust prediction power and strong generalization ability of NPCDS were proven within each independent cohort, which largely benefits from our meticulously designed framework. Our NPCDS, composed of fewer but divergent features (nine neutrophil-derived PCD genes), was generated via a combination of RF and Stepglm, and therefore has a superior extrapolation possibility. To further extend the clinical application of NPCDS, an external cohort with clinical characteristics was included for verification. And the robust predictive performance of NPCDS was again proven. We then integrated AMI-related clinical characteristics (Betablockers, ACEI, Diabetes status, and Statins) and NPCDS scores to develop a nomogram. The desirable predictive power and clinical practicability of the nomograph were demonstrated following the assessment criteria of AUC, calibration, and DCA. Leveraging the graphic-visualized nomograph, a clinician could easily compute an individual’s specific probability of AMI, which can be stratified (low, medium, and high risk). Interestingly, our nomograph incorporating NPCDS and clinical characteristics could improve prediction performance compared to sole NPCDS usage and, more preferably, clinical decisions for medium-risk or high-risk patients.

Given the heterogeneity of AMI, we also wondered whether NPCDS could serve as a novel molecular subtype, which may contribute to stratified therapy developed for AMI patients. Herein, the NMF algorithm allows for the stable separation of AMI patients into two divergent subtypes, which were verified in multiple cohorts. To gain more detailed insights into molecular characteristics that differed in subtypes, we then measured the enrichment of PCD patterns and neutrophil activities within the two subtypes. Interestingly, our established subtypes satisfactorily captured distinct immunological statuses of AMI, in which subtype 1 represents immunoactivation viability with vibrant patterns of NETosis, neutrophil, neutrophil degranulation, and neutrophil extravasation; whereas subtype 2 stands for immunosuppression with inhibited patterns. GSEA was subsequently implemented to uncover that the positive abundance of NETosis, neutrophil, and neutrophil degranulation significantly reached in subtype 1 compared to subtype 2. Previous work has correlated these elevated neutrophil processes with adverse cardiac events post-AMI [74, 75]. Therefore, subtype 1 may be more prone to poor clinical outcomes, indicating the neutrophils’ therapeutic implications for AMI. Our findings support the promising clinical utility of the NPCDS-derived molecular subtype of AMI.

Of the nine genes enrolled in developing NPCDS, Protein Tyrosine Kinase 2 Beta (PTK2B) encodes a cytoplasmic protein tyrosine kinase, was discovered to be significantly activated in non-ischemic dilated cardiomyopathy [76]. Notably, inhibition of PTK2B has been proven to improve cardiac function in rat models with heart failure [77] and post-AMI ventricular remodeling [78]. Mitogen-Activated Protein Kinase 14 (MAPK14), encoding the most abundant α-isoform of p38 MAPK in human cardiac tissue, has been found to aggravate myocardial infarction during ischemi [79]. Nevertheless, the cardioprotective role of MAPK14 was disclosed through the degression of ischemic load post-AMI [80], which suggested its controversial double impact on AMI. In mice with MAPK14 overexpression, the heart exhibits interstitial and perivascular fibrosis due to an increase in the number of myofibroblasts, whereas the absence of MAPK14 prevents cardiac fibroblasts from differentiating into myofibroblasts in response to ischemic injury, thereby hindering subsequent fibrosis [81]. Influenza Virus NS1A Binding Protein (IVNS1AB) encodes an actin-binding protein ND1 to prevent actin derangement [69], which plays a fundamental role in the locomotion and phagocytosis of neutrophils [82]. IVNS1AB overexpression in mouse hearts reduces cardiomyocyte apoptosis, protecting against doxorubicin-induced cardiomyopathy [83]. Deficiency of IVNS1AB in mouse embryonic fibroblasts increases cardiomyocyte apoptosis when exposed to doxorubicin [84]. Myosin Heavy Chain 9 (MYH9) encodes non-muscle myosin heavy chain IIA [85], and its abnormality in the hematopoietic system alters platelet cytoskeletal components, leading to bleeding disorder [86]. Myeloid Differentiation factor 88 (MYD88), an intracellular adaptor protein, plays a pivotal role in coordinating pro-inflammatory signaling pathways [87]. MYD88 absence in mice could improve post-MI survival and reduce cardiac hypertrophy [88]. Additionally, MYH9 and MYD88 were shown to regulate neutrophil locomotion [89], such as crawling, migration, and extravasation. Toll Like Receptor 2 (TLR2), a highly conserved member of the TLR family, has been implicated in modulating post-AMI ventricular remodeling [90]. Beneficial cardioprotective effects after inhibition of TLR2, such as preservation of cardiac function and attenuation of ischemia reperfusion injury, were observed in the murine model with AMI [90, 91]. Glucosamine (*N*-Acetyl)-6-Sulfatase (GNS), also known as G6S, encodes a marker lysosomal enzyme reflecting lysosome involvement in AMI [92]. Nevertheless, insufficient work exploring the functions of GNS on AMI or neutrophil activities. CASP8 and FADD Like Apoptosis Regulator (CFLAR) and E3 Ubiquitin-Protein Ligase MDM2 have long been regarded as anti-apoptotic molecules that receptively suppress caspase-8 [93] and pro-apoptotic transcription factor p53 [94]. In view of the prompt clearance of neutrophils to limit inflammation and injury post-AMI, we speculated that the inhibition of anti-apoptotic regulators (CFLAR and MDM2) could re-initiate the apoptotic program, contributing to enhancing the cardiac functions post-AMI. In post-myocardial infarction rodents, reduced CFLAR expression is observed in the ventricular myocardium, while CFLAR knockout mice display impaired cardiac trabeculae formation and myocardial thinning [95]. Of note, MDM2 inhibition was recently demonstrated to alleviate cardiac dysfunction and fibrosis in a murine model with AMI [96]. Taken together, the present studies have revealed the close relationships between these genes and AMI pathogenic or neutrophil processes. Nevertheless, their complicated interplays, particularly how these PCD genes modulate the neutrophil activities during AMI progression, remain obscure. We attempt to bridge this knowledge gap in subsequent analysis.

ScRNA-seq provides an ideal approach to mapping the immune cell landscape in AMI and can contribute to exploring molecular characteristics and dynamics [97]. We herein implemented scRNA-seq analysis on Cd45+ immune cells isolated from infracted murine hearts post-AMI. We identified six putative clusters that were annotated into five cell populations, leveraging previously reported marker genes [26], including macrophage/monocyte, T/NK cell, neutrophil, B cell, and dendritic cell. It is noteworthy that neutrophils increased sharply from day 3 to day 7 post-AMI but plummeted on day 14, which agrees with prior studies using animal models [17]. We next analyzed neutrophil dynamics and detected two critical subpopulations: N1 elevated on day 3 post-AMI but nearly lost on day 7, identified with pro-inflammatory function; and N2 raised on day 7 post-AMI, identified with anti-inflammatory function. Recent studies have suggested the presence of neutrophils with proinflammatory and anti-inflammatory phenotypes in infracted hearts following AMI [19, 26], which supported our identification of neutrophil subpopulations. NPCDS showed differential expression patterns among the two subpopulations of neutrophils, in which N1 significantly expresses Myd88 and Myh9, whereas N2 significantly expresses Cflar and Mdm2. Pseudotime analysis was leveraged on the neutrophil population to infer the transition trajectory from N1 to N2. We then clustered NPCDS into divergent kinetic patterns across the neutrophil transition, with Mapk14, Myd88, Myh9, Ptk2b, and Tlr2 as lower pseudotime-related patterns corresponding to N1 status; Gns, Mdm2, and Cflar as higher pseudotime-related patterns corresponding to N2 status. As a result, we unveiled the dynamic role of NPCDS in modulating the neutrophil conversion from the pro-inflammatory phase (N1 subtype with lower pseudotime) to the anti-inflammatory phase (N2 subtype with higher pseudotime) in the progression of AMI, which broadened our understanding of NPCDS.

To fully elucidate the mechanism of NPCDS, we retrieved the KEGG database and carried out Bayesian network inference. We characterized the tight interplays between NPCDS-dominated pathways, including NETosis, apoptosis, neutrophil migration, and LDCD. We found that CFALR, which displayed a significant negative coefficient in the NPCDS model, serves as a downstream suppressor of apoptosis. More intriguingly, we noticed that the genes highly expressed in the early stage of neutrophil conversion (TLR2, MAPK14, MYD88, PTK2B, and MYH9) appeared as upstream in the pathways, whereas those delayal elevated in the late stage (MDM2, CFALR, and GNS) stand for the downstream roles. For instance, the initiation of apoptosis results from the early stimulation of TLR2 triggering MYD88 activity, which engages FADD for the binding and activation of caspase 8; while upregulations of CFLAR and MDM2 in the late stage induce the suppression of caspase 8. In summary, we first elucidated the dynamic expressions of NPCDS during neutrophil transformation in AMI from scRNA-seq analysis. Then, we integrated the established pathway information to reveal the latent molecular mechanisms of NPCDS governing the transformation. Of note, our findings suggest that the activations of NETosis, apoptosis, neutrophil migration, and LDCD regulated via NPCDS support the N2 conversion to initiate cell death and attenuate inflammation status.

Therapeutic manipulation of neutrophil lifespan, particularly targeting the molecular mechanisms of cell death, has been successfully shown to resolve inflammation in preclinical models [20, 98]. We previously characterized apoptosis as a crucial molecular mechanism underlying NPCDS that facilitates the transition from N1 to N2, among which MDM2 and CFALR are downstream suppressor regulators. Given that delay in apoptosis allows the impaired clearance of dead neutrophils [99], we hypothesized that the contribution of MDM2 and CFALR to suppress apoptotic processes hampers the timely resolution of inflammation. Herein, we report the significant findings from two-sample MR analysis and drug prediction on MDM2. After performing MR analysis on GWAS datasets, we found a positive causal relationship between elevated MDM2 expression and an increased risk of AMI. Involvement of MDM2 has previously been proven in myocardial ischemic injury, and MDM2 inhibition could attenuate cardiac dysfunction post-AMI [96]. After myocardial infarction, the upregulation of MDM2 protein expression in mouse hearts is significantly attenuated by treatment with the MDM2 inhibitor Nutlin-3a, which inhibits NF-κB activation, blocks pro-inflammatory cytokine production, and improves cardiac function while reducing cardiac fibrosis. Despite these findings from the myocardium, MDM2 is potentially of great importance in controlling neutrophil vitality due to its suppression of apoptosis. Therefore, targeting MDM2 may represent a potential efficacy for treating AMI [100]. Based on the Enrichr database, three candidate small-molecule drugs, including lidoflazine, isotetrandrine (Fig. 9F), and cepharanthine were predicted to target MDM2. Among these predictive drugs, lidoflazine, an antianginal calcium channel blocker, has been proven to show myocardial protective effects [101]. Moreover, the anti-inflammatory properties of isotetrandrine and cepharanthine have been investigated [102, 103], suggesting potential pharmaceutical effectiveness against myocardial injury. Using molecular docking to measure affinity, we found stable bindings of these drugs with MDM2 via the generation of hydrogen bonds, which demonstrates the druggability of MDM2. We next integrated GSEA and ssGSEA algorithms to analyze an in-vivo experimental cohort, which includes RNA-seq datasets on heart specimens isolated from Mdm2-knockout mice, to delve deeper into understanding how Mdm2 modulates neutrophil activities. Intriguingly, we found that Mmd2 deficiency contributes to the elevated enrichment of p53 signaling, resulting in the enhancement of apoptosis and suppression of the activation process. Given MDM2’s role in curbing the resolution of inflammation in AMI, inhibition of MDM2 would appear to a prompt clearance of aggregated neutrophils after AMI. Overall, we provide information for understanding MDM2 as a pathogenic and therapeutic target of AMI, which may contribute to developing a novel molecular therapy.

Though the potential implications of NPCDS in predicting, stratifying, and treating AMI were demonstrated, several limitations in our work should be noted. First, the samples enrolled in this study were retrospective and inadequate, and verification should be further conducted within a prospective and larger multi-center collaboration, which could guarantee extensive application of NPCDS. Second, the clinical traits were insufficient to comprehensively reflect the pathophysiologic status of AMI, which minorly contributed to developing a robust nomogram. More detailed clinical traits should be included in further investigation. Third, the specific biological mechanism underlying NPCDS across the neutrophil transition was still worth confirmation and elucidation through more in-vivo and in-vitro experiments. Finally, MDM2 as a molecular therapeutic target of AMI warrants genetic knockout and pharmacological exploration in animal models.

## Conclusion

Our work provides a neutrophil-derived programmed cell death signature (abbreviated NPCDS) that exhibited superior performance of AMI prediction within eight independent cohorts from peripheral blood. Our integrative machine learning-based framework generated NPCDS, which is a combination of RF and Stepglm fit on a nine-gene panel (MDM2, PTK2B, MYH9, IVNS1ABP, MAPK14, GNS, MYD88, TLR2, CFLAR). Additionally, we leveraged NPCDS to develop a novel molecular subtype of patients with AMI, with subtype 1 of higher neutrophil activities being more prone to adverse clinical outcomes. Of importance, we uncovered the biological mechanism underlying NPCDS across the neutrophil conversion from a pro-inflammatory to an anti-inflammatory state. Through MR analysis, we found a positive causal association between elevated MDM2 expression and AMI risk. We also revealed the druggable value of MDM2 in curing AMI, potentially in the resolution of inflammation through triggering neutrophil apoptosis. Overall, our NPCDS model has great potential in the diagnosis, stratification, and treatment of patients with AMI in clinical applications.

### Supplementary Information

Supplementary Material 1. Supplementary Table 1. PCD-related gene sets. Supplementary Table 2. Neutrophil-related gene sets. Supplementary Table 3. The normalized ssGSEA scores of 17 PCD-related and Neutrophil-related gene sets. Supplementary Table 4. SOM on expression profile of training set. Supplementary Table 5. Detailed information of DEGs analysis of training set. Supplementary Table 6. The Pearson’s correlation analysis on Neutrophil-related and PCD-related genes. Supplementary Table 7. A total of 63 published signatures were retrieved from the literatures. Supplementary Table 8. Clinical information of external validation set (GSE34198). Supplementary Table 9. NMF results of GSE123342, GSE60993, GSE34198. Supplementary Table 10. GSEA on cluster 1 and cluster 2 of GSE123342, GSE60993, GSE34198 using NETosis, neutrophil, and neutrophil degranulation gene sets. Supplementary Table 11. Percentage of cell population at different periods in control and experimental groups. Supplementary Table 12. Percentage of neutrophil population at different periods in control and experimental groups. Supplementary Table 13. Within Clusters Sum of Squares (WCSS) metric was utilized as a basis to determine the optimal numbers of K-means clustering on DEGs between N1 and N2 subgroups. Supplementary Table 14. Sensitivity analysis of two-sample MR. Supplementary Table 15. Predictive drugs targeting MDM2 via Enrichr database.

Supplementary Material 2. Supplementary Figure 1. Training progress of performing SOM on expression profile of training set. (A) Stable SOM algorithm was implemented through 100 iterations. (B) WCSS metric was utilized as a basis to decide the optimal numbers of K-Means clustering on SOM nodes.

Supplementary Material 3. Supplementary Figure 2. External validation of NPCDS within GSE48060 and GSE194388. (A) Left panel: Overview of control and AMI samples utilized in GSE48060 and confusion matrix. Right panel: ROC analysis of NPCDS in GSE48060. (B) Left panel: Overview of control and AMI samples utilized in GSE194388 and confusion matrix. Right panel: ROC analysis of NPCDS in GSE194388. (C) Comparison of NPCDS and published gene expression signatures in GSE48060 and GSE194388.

Supplementary Material 4. Supplementary Figure 3. Heatmap displaying the distribution of clinical characteristics among two clustering patterns in GSE34198.

Supplementary Material 5. Supplementary Figure 4. Quality control and dimensional reduction on scRNA-seq data. (A) Filtering out cells with less than 200 or larger than 5000 feature counts and mitochondrial proportion larger than 20%. (B) Correlation between gene counts and feature counts. (C) Identification of highly variable genes. The red dots represent the top 2000 highly variable genes and the top 10 labeled with gene symbols. (D) The 20 principal components (PCs) on scaling data and 30 representative genes in each PC. (E) Heatmaps of representative genes in each PC. (F) PCA score plot of data using two PCs. (G) Elbow plot ranking PCs on the percentage of variance indicated the top 15 PCs were determined for cell clustering.

Supplementary Material 6. Supplementary Figure 5. Annotation on clusters using specific gene markers. (A) Cd45 (Ptprc) expression among clusters, and clusters 5 and 7 showed with low Cd45 expression were removed. (B) Expression of Macrophage/Monocyte gene markers among clusters. (C) Expression of T/NK gene markers among clusters. (D) Expression of neutrophil gene markers among clusters. (E) Expression of B cell gene markers among clusters. (F) Expression of dendrite cell gene markers among clusters. (G) Dot plot showing the relative expression levels of marker genes among the five annotated cell types.

Supplementary Material 7. Supplementary Figure 6. Annotation on sub-clusters of neutrophils using inflammatory, anti-inflammatory, and apoptosis gene markers.

Supplementary Material 8. Supplementary Figure 7. Pseudotime inference analysis of feature genes. (A) Expressions of feature among Pseudotime-ordered neutrophil populations. (B) WCSS metric was utilized as a basis to decide the optimal numbers of K-Means clustering on DEGs between N1 and N2 sub-populations.

Supplementary Material 9. Supplementary Figure 8. The locations of feature genes in the KEGG pathways. (A) TLR2 and MAPK14 in Neutrophil extracellular trap formation. (B) TLR2, MYD88, and MAPK14 in Toll-like receptor signaling pathway. (C) MDM2 in p53 signaling pathway. (D) CFLAR in Apoptosis. (E) GNS in Lysosome. (F) PTK2B and MYH9 in Leukocyte transendothelial migration.

## Data Availability

The GEO databases (http://www.ncbi.nlm.nih.gov/geo) contain the datasets (GSE123342, GSE29532, GSE60993, GSE61144, GSE97320, GSE48060, GSE194388, GSE34198, GSE16346, GSE151571) used to support this study.

## Abbreviations

PCD: Programmed cell death
AMI: Acute myocardial infarction
NPCDS: Neutrophils-derived PCD signature
MR: Mendelian randomization
CAD: Coronary artery disease
cTn: Cardiac troponin
ESC: European Society of Cardiology
ACC: American College of Cardiology
ACS: Acute coronary syndrome
ROS: Reactive oxygen species
ML: Machine learning
NMF: Non-negative matrix factorization
scRNA-seq: Single-cell RNA sequencing
ssGSEA: Single sample Gene Set Enrichment Analysis
SOM: Self-organizing map
DEGs: Differentially expressed genes
FC: Fold change
FDR: False discovery rate
DO: Disease Ontology
GO: Gene Ontology
KEGG: Kyoto Encyclopedia of Genes and Genomes
Lasso: Least Absolute Shrinkage and Selection Operator
Enet: Elastic network
Stepglm: Stepwise generalized linear model
SVM: Support Vector Machine
glmBoost: Generalized linear model by likelihood-based Boosting
LDA: Linear discriminant analysis
plsRglm: Partial least squares regression for generalized linear models
RF: Random Forest
GBM: Gradient boosting machine
XGBoost: EXtreme Gradient Boosting
CV: Cross-Validation
AUC: Area Under Curve
DCA: Decision Curve Analysis
PCA: Principal component analysis
PCs: Principal components
t-SNE: T-distributed Stochastic Neighbor Embedding
BN: Bayesian Network
GRN: Gene Regulatory Network
SNPs: Single Nucleotide Polymorphisms
IVs: Instrumental variables
LD: Linkage disequilibrium
IVW: Inverse variance weighted
OR: Odds ratio
RMSE: Root Mean Squared Error
ROC: Receiver operating characteristic curve
LOOCV: Leave-One-Out Cross-Validation
WCSS: Within Clusters Sum of Squares
PTK2B: Protein Tyrosine Kinase 2 Beta
MAPK14: Mitogen-Activated Protein Kinase 14
IVNS1ABP: Influenza Virus NS1A Binding Protein
MDM2: Mouse Double Minute 2 homolog
GNS: Glucosamine (*N*-Acetyl)-6-Sulfatase
MYH9: Myosin Heavy Chain 9
TLR2: Toll Like Receptor 2
MYD88: Myeloid Differentiation factor 88
CFLAR: CASP8 and FADD Like Apoptosis Regulator
