# Supplementary figures and images for: Leveraging a neutrophil-derived PCD signature to predict and stratify patients with acute myocardial infarction: from AI prediction to biological interpretation
Source: J Transl Med. 2024 Jul 2;22:612. doi: 10.1186/s12967-024-05415-0 (PMC11221097; doi:10.1186/s12967-024-05415-0)

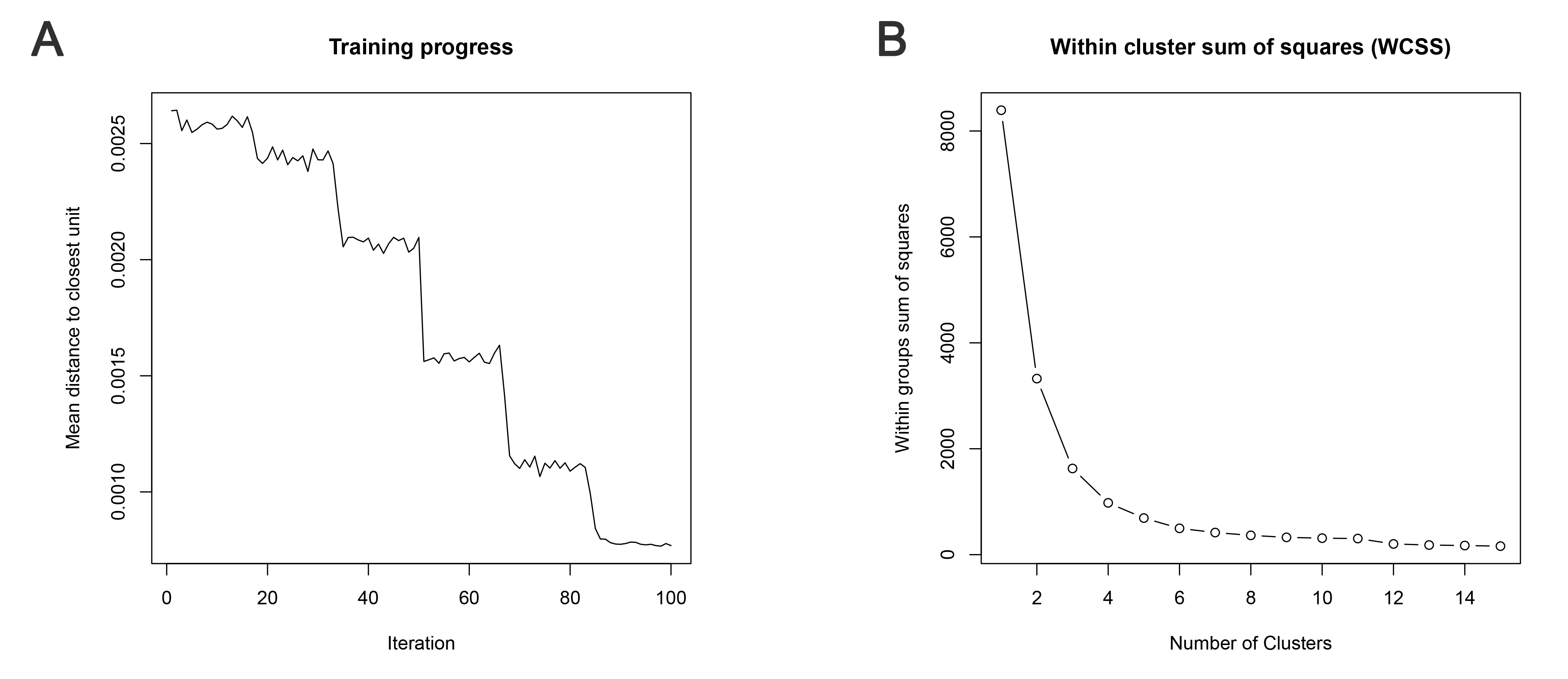

Supplement: Supplementary file 2 — Supplementary Material 2. Supplementary Figure 1. Training progress of performing SOM on expression profile of training set. (A) Stable SOM algorithm was implemented through 100 iterations. (B) WCSS metric was utilized as a basis to decide the optimal numbers of K-Means clustering on SOM nodes. [file 12967_2024_5415_MOESM2_ESM.jpg]

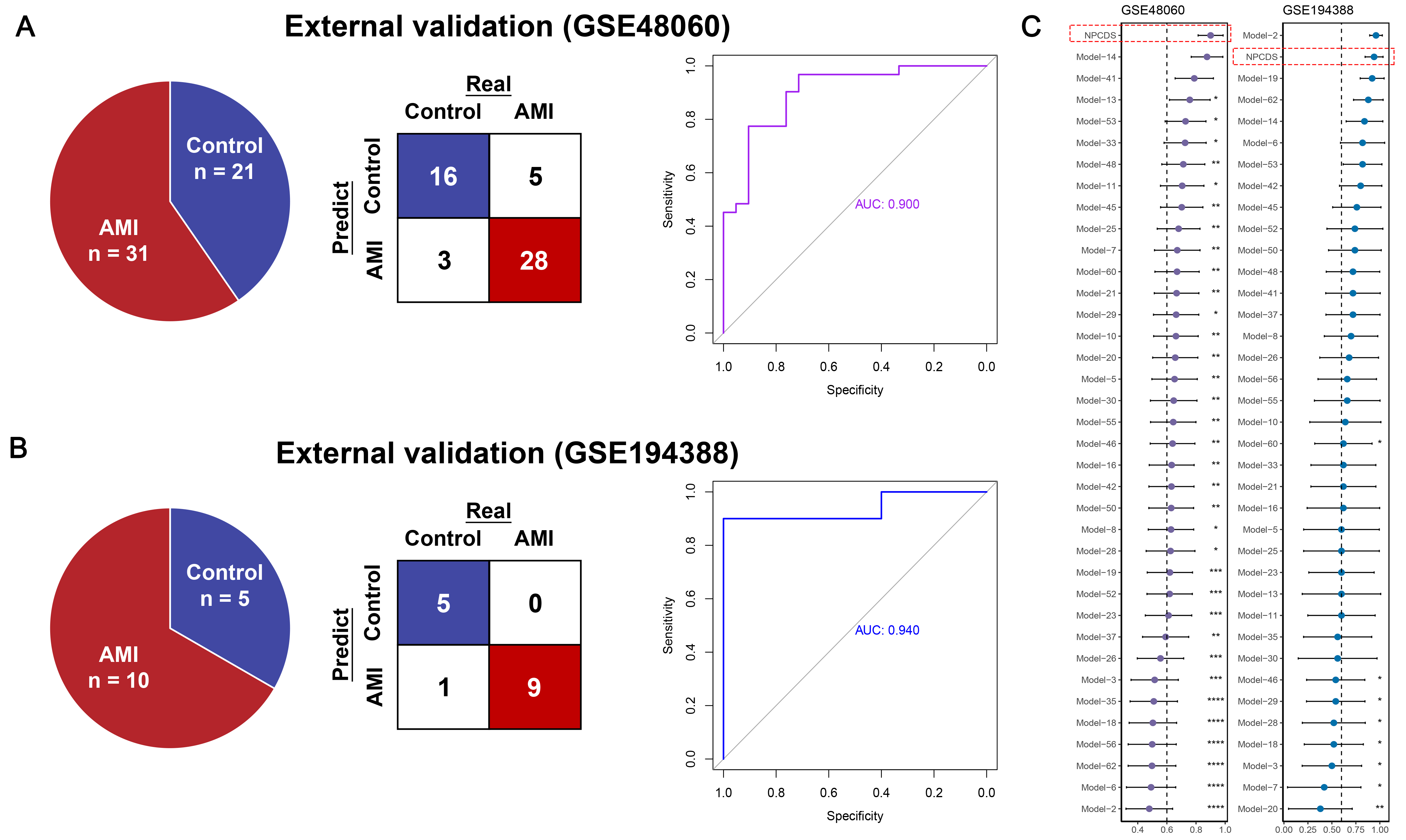

Supplement: Supplementary file 3 — Supplementary Material 3. Supplementary Figure 2. External validation of NPCDS within GSE48060 and GSE194388. (A) Left panel: Overview of control and AMI samples utilized in GSE48060 and confusion matrix. Right panel: ROC analysis of NPCDS in GSE48060. (B) Left panel: Overview of control and AMI samples utilized in GSE194388 and confusion matrix. Right panel: ROC analysis of NPCDS in GSE194388. (C) Comparison of NPCDS and published gene expression signatures in GSE48060 and GSE194388. [file 12967_2024_5415_MOESM3_ESM.jpg]

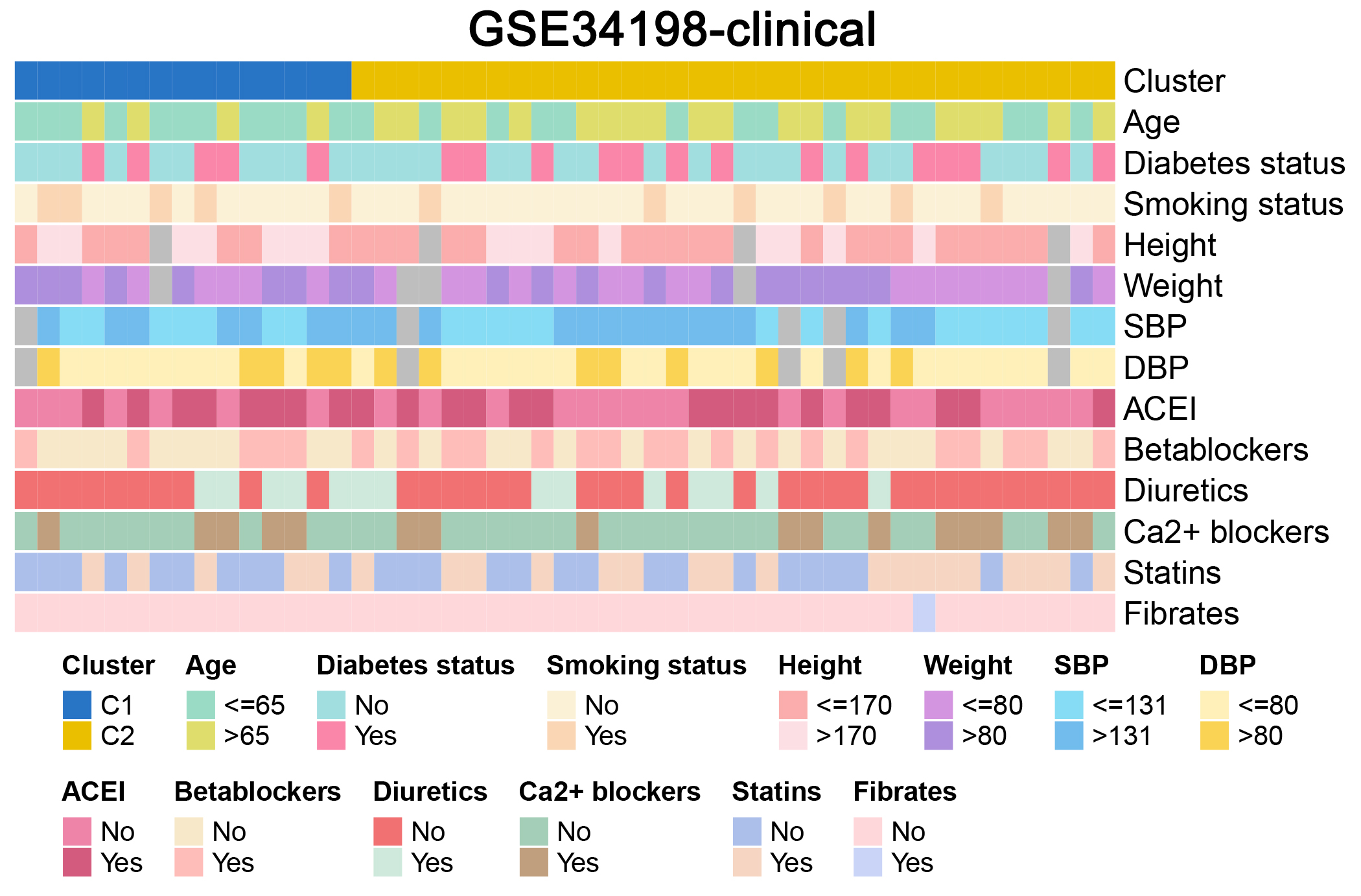

Supplement: Supplementary file 4 — Supplementary Material 4. Supplementary Figure 3. Heatmap displaying the distribution of clinical characteristics among two clustering patterns in GSE34198. [file 12967_2024_5415_MOESM4_ESM.jpg]

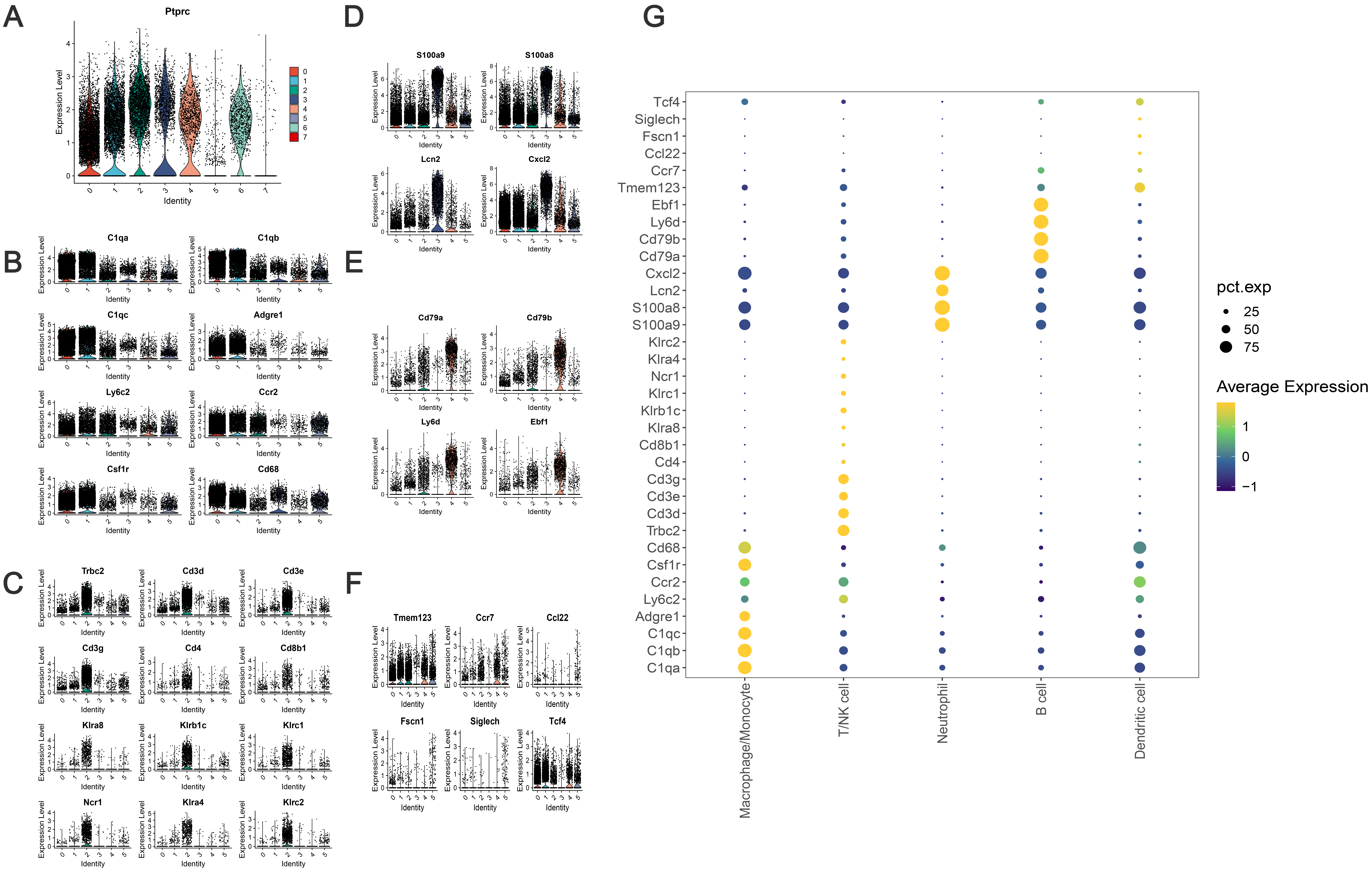

Supplement: Supplementary file 6 — Supplementary Material 6. Supplementary Figure 5. Annotation on clusters using specific gene markers. (A) Cd45 (Ptprc) expression among clusters, and clusters 5 and 7 showed with low Cd45 expression were removed. (B) Expression of Macrophage/Monocyte gene markers among clusters. (C) Expression of T/NK gene markers among clusters. (D) Expression of neutrophil gene markers among clusters. (E) Expression of B cell gene markers among clusters. (F) Expression of dendrite cell gene markers among clusters. (G) Dot plot showing the relative expression levels of marker genes among the five annotated cell types. [file 12967_2024_5415_MOESM6_ESM.jpg]

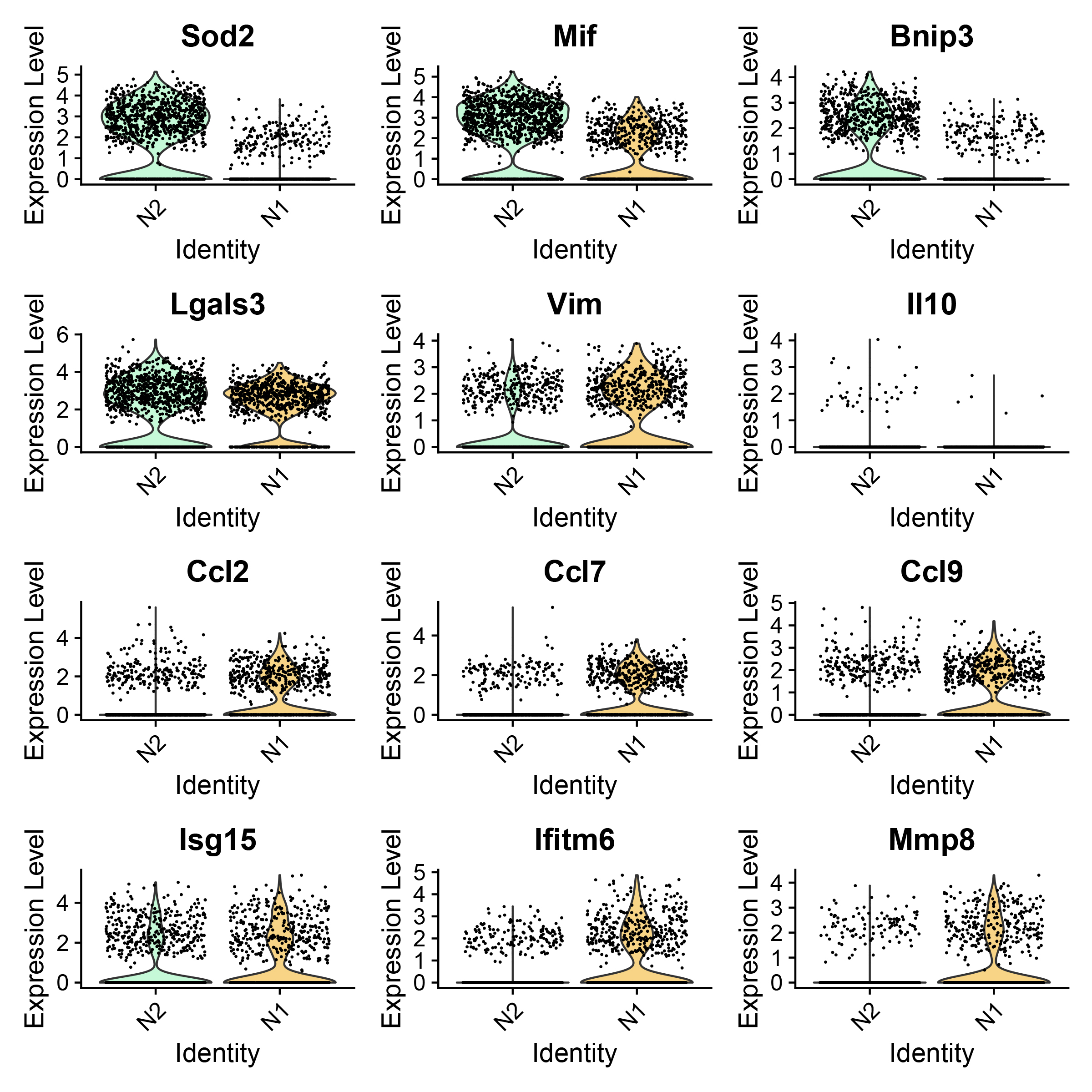

Supplement: Supplementary file 7 — Supplementary Material 7. Supplementary Figure 6. Annotation on sub-clusters of neutrophils using inflammatory, anti-inflammatory, and apoptosis gene markers. [file 12967_2024_5415_MOESM7_ESM.jpg]

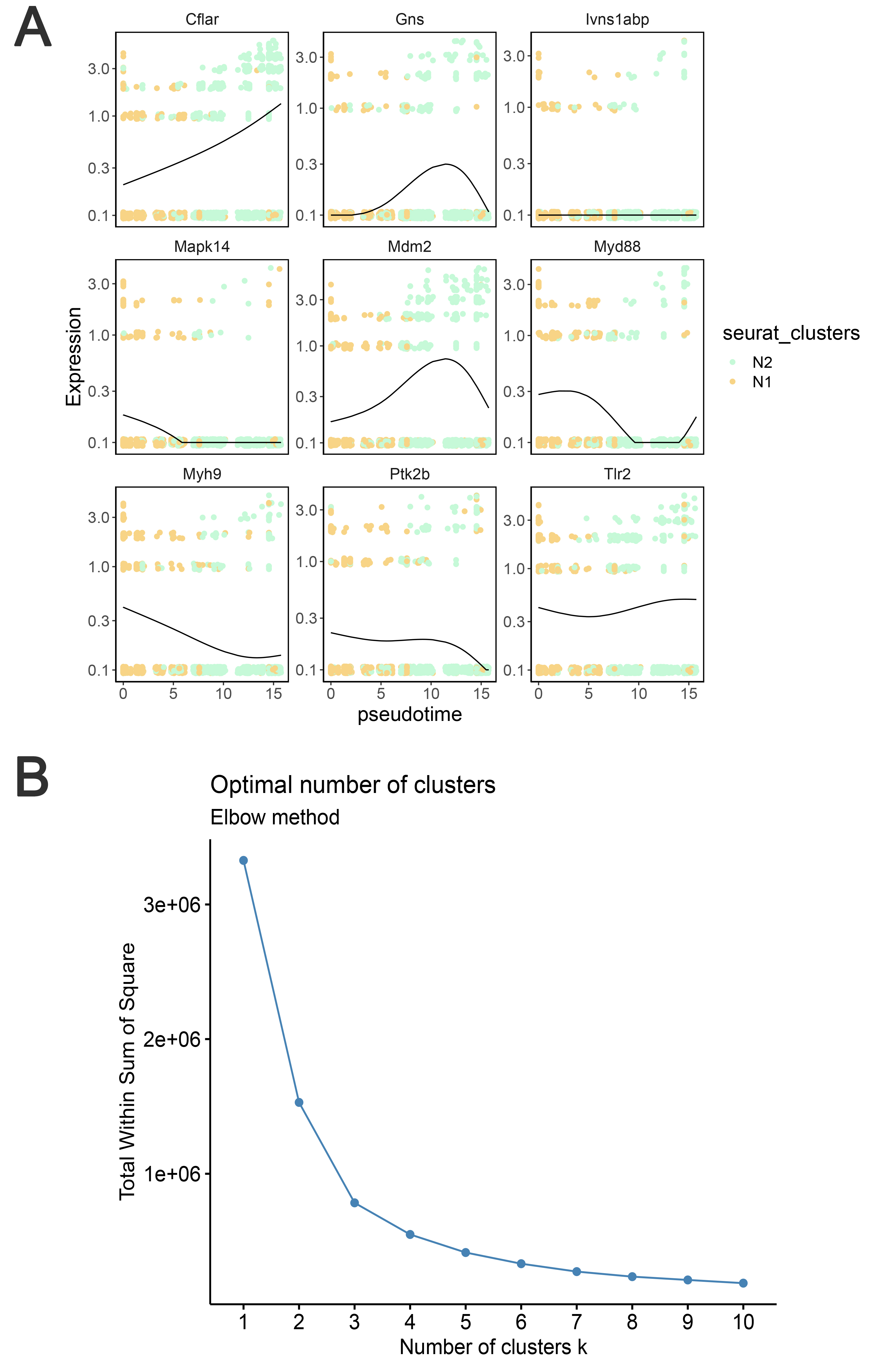

Supplement: Supplementary file 8 — Supplementary Material 8. Supplementary Figure 7. Pseudotime inference analysis of feature genes. (A) Expressions of feature among Pseudotime-ordered neutrophil populations. (B) WCSS metric was utilized as a basis to decide the optimal numbers of K-Means clustering on DEGs between N1 and N2 sub-populations. [file 12967_2024_5415_MOESM8_ESM.jpg]
